# Blocking PD1 prevents T-cell-promoted fibrosis in myocardial infarction

**DOI:** 10.1038/s41421-026-00924-2

**Published:** 2026-10-06

**Authors:** Xiaoying Chen, Changchen Xiao, Changle Ke, Jiamin Li, Ye Liu, Junhua He, Mo Li, Jing Zhao, Zhiwei Zhong, Qiming Chen, Tingting Hong, Fei Liao, Feimu Zhang, Yu Zhang, Jiayue Cai, Shuyuan Sheng, Qingju Li, Yongjian Chen, Yinghui Xu, Jingyi Wang, Shuhan Zhong, Xianpeng Wu, Peng Shi, Yang Xu, Wei Zhu, Xinyang Hu, Cheng Ni, Jian’an Wang

**Affiliations:** 1 Department of Cardiology of The Second Affiliated Hospital, School of Medicine, Zhejiang University, Hangzhou, Zhejiang China; 2 State Key Laboratory of Transvascular Implantation Devices, Hangzhou, Zhejiang China; 3 Heart Regeneration and Repair Key Laboratory of Zhejiang Province, Hangzhou, Zhejiang China; 4 Binjiang Institute of Zhejiang University, Hangzhou, Zhejiang China; 5 Transvascular Implant Instrument Research Institute, The Second Affiliated Hospital Zhejiang University School of Medicine, Hangzhou, Zhejiang China

**Keywords:** Tumour immunology, Cell biology

## Abstract

Myocardial fibrosis is a common pathological feature that affects the outcome of various heart diseases, typically in the context of myocardial infarction (MI). The role of T cells in heart failure has been increasingly recognized, but whether programmed cell death protein 1 (PD1^+^) T cells modulate cardiac fibrosis during the post-MI pathological remodeling process remains unclear. Single-cell RNA sequencing and mass cytometry were conducted to determine the proportion of PD1^+^ T cells in both human ischemic diseases and a mouse MI model. Bulk RNA sequencing and cytokine arrays were used to investigate the function of PD1^+^ T cells. Cardiac function and histology were evaluated in non-human primates and rodents post-MI. We observed significant enrichment of PD1^+^ T cells in the heart after MI, which was positively associated with cardiac fibroblast activation and, hence, collagen secretion. Unlike in tumors, PD1^+^ T cells in the heart after MI demonstrated activated characteristics. PD1 knockout mice exhibited reduced cardiac fibrosis, resulting in increased cardiac performance following MI. Mechanistically, activated PD1^+^ T cells were found to drive fibrosis remodeling by modulating the CXCL9/CXCR3 axis through direct interaction with cardiac fibroblasts — an effect that occurred independently of the PD1/programmed death-ligand 1 (PD-L1) signaling pathway. Notably, anti-PD1 therapy in both mouse and non-human primate MI models effectively attenuated fibrosis and improved cardiac function without eliciting detectable adverse effects in non-cardiac organs. Our findings reveal a distinct PD-L1-independent pro-fibrotic mechanism mediated by PD1^+^ T cells during post-MI cardiac remodeling. Therapeutic inhibition of PD1^+^ T cells effectively suppressed fibrosis progression, highlighting its potential as an immunotherapeutic target for post-MI cardiac repair.

## Introduction

Myocardial fibrosis is a common pathological consequence of nearly all forms of heart disease, most notably following myocardial infarction (MI), and serves as a major risk factor for predicting heart failure outcomes^1^. It is widely recognized that adverse cardiac remodeling, particularly fibrosis, is driven by interactions among multiple cell types and cardiac fibroblasts following MI. However, the detailed molecular mechanisms remain unclear, and robust supporting evidence is lacking. Therefore, deciphering the complex cellular crosstalk within the heart under pathological circumstances is imperative, as it holds the key to the development of innovative strategies to combat fibrosis.

Emerging evidence underscores the critical involvement of inflammatory and immune processes in mediating myocardial injury, orchestrating infarct healing, and influencing subsequent ventricular remodeling^2^. In the acute phase of MI, distinct types of inflammatory cells are sequentially recruited to the infarcted area to facilitate necrotic tissue clearance and scar formation^3^. As pivotal components of adaptive immunity, T cells regulate fibroblast function and related biological behavior during the post-MI cardiac remodeling process^4,5^. It has been shown that T-cell activation also aids in necrotic substance clearance and promotes cardiac repair in the acute-MI phase; however, prolonged T-cell responses in the infarcted heart can lead to adverse remodeling and contribute to the progression of cardiac fibrosis^6^. Precise temporal and spatial regulation of these biphasic immune cell populations is essential for maintaining reparative processes. It is hoped that data produced from studies focused on T cells may elucidate novel reparative mechanisms and provide potential therapeutic targets for translation to clinical patients with MI.

Programmed cell death protein 1 (PD1) is crucial for adaptive immune regulation and has considerable effects on various diseases, especially tumors. With respect to the canonical function of PD1, programmed death-ligand 1 (PD-L1) on cell surfaces binds to PD1, inhibits T-cell activation, induces apoptosis, and incapacitates cells^7^. However, under inflammatory conditions such as rheumatoid arthritis, PD1^hi^CD4^+^ T cells exhibit a proinflammatory function with increased expression of the cytokines TNF-α, IFN-γ, and IL-2^8^. In patients with acute COVID-19, PD1^+^ T cells exhibit proliferative function and strongly express the activation marker CD38^9^. This intricate regulatory role of PD1 on T cells also highlights its involvement in MI. Nonetheless, the function of PD1^+^ T cells in the heart remains unclear and requires further investigation.

In the present study, we revealed that PD1^+^ T cells play a significant role in promoting adverse cardiac remodeling and myocardial fibrosis post-MI. Surprisingly, PD1^+^ T cells exhibited distinct immunological behavior in MI models compared to those in tumors models, which showed activated immune-regulatory characteristics. In addition, CXCL9 in PD1^+^ T cells was shown to be the pivotal target for activating cardiac fibroblasts via the interaction of CXCR3, and this biological process was independent of the canonical PD1/PD-L1 interaction. The therapeutic effects of anti-PD1 therapy via monoclonal antibody in salvaging cardiac function by alleviating cardiac fibrosis following MI have been validated in both rodent and non-human primate MI models, providing important hints regarding its translational prospects for treating MI in the clinic.

## Results

### MI drives a pronounced expansion of PD1^+^ T cells

**Fig. 1:**
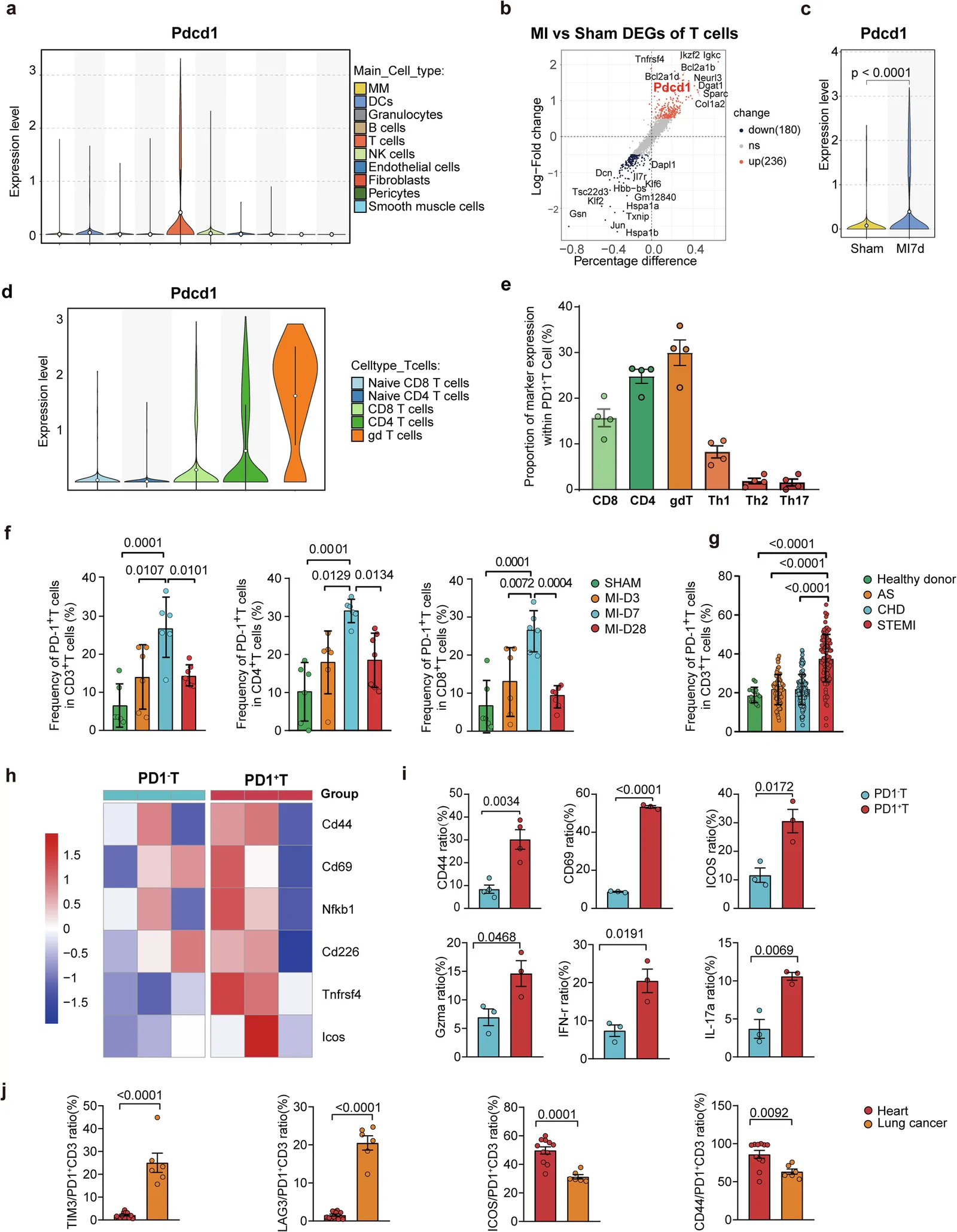
PD1^+^ T cells are enriched and activated in the infarcted mouse heart. **a** Violin plot of *Pdcd1* (encoding PD1) expression in different types of non-myocytes in hearts subjected to MI surgery. **b** Volcano plot of genes upregulated in cells isolated from MI hearts compared with those isolated from sham hearts at 7 days post-MI; log_2_FC > 0.5, *P*-value_adj < 0.05. **c** Comparison of *Pdcd1* gene expression between MI hearts and sham hearts. **d** scRNA-seq analysis of CD3^+^ T cells isolated from murine hearts 7 days post-MI. Violin plots depict m*Pdcd1* expression levels across six major T-cell subsets: naive CD8^+^ T cells, naive CD4^+^ T cells, effector CD8^+^ T cells, effector CD4^+^ T cells, Tregs, and γδ T cells. **e** Flow cytometry-mediated quantification of PD1 expression levels across different T-cell subsets in murine hearts 7 days post-MI. **f** Flow cytometry results showing the percentage of PD1^+^ cells among CD3^+^ T cells, CD4^+^ T cells^,^ and CD8^+^ T cells in the mouse heart at 3, 7, and 28 days after MI; *n* = 6–7 in each group. **g** Flow cytometry results showing the percentage of PD1 expression on CD3^+^ T cells in the peripheral blood of healthy donor (*n* = 14), AS (*n* = 62), CHD (*n* = 116), and STEMI patients (*n* = 90). **h** Heatmap of genes associated with upregulated T-cell activation in PD1^+^ T cells vs PD1^−^ T cells. **i** Flow cytometry analysis showing the percentage of activation marker (CD44, CD69, and ICOS)-positive cells among PD1^+^ T cells and PD1^−^ T cells 7 days after MI; *n* = 34 mice per group. **j** Flow cytometry analysis showing the PD1 level in the infarcted heart on Day 7 and after subcutaneous transplantation of tumors with Lewis cells, as well as exhausted (TIM3, LAG3) and activated (ICOS, CD44) T-cell markers in PD1^+^ T cells (infarcted heart, *n* = 11; tumor tissue, *n* = 6). Comparisons between cohorts were performed using an unpaired two-tailed Student’s *t*-test. Multiple group comparisons were performed using one-way ANOVA with Tukey’s post hoc test. The bars indicate the SEMs.

To investigate the expression characteristics of PD1^+^ T cells in the heart during MI in depth, we first performed single-cell RNA sequencing (scRNA-seq) to characterize non-cardiomyocytes from mice after MI (Supplementary Fig. S1a, b). Among all the examined cell types, T cells displayed the highest PD1 expression on Day 7 after MI (Fig. 1a). The expression level of PD1 on T cells was significantly greater in the MI group than in the sham group (Fig. 1b, c). On the basis of lineage-specific gene expression signatures, six distinct T-cell clusters were identified (totaling 2118 cells), among which the proportion of PD1^+^ T cells markedly increased from 4.97% to 22.02% after MI (Supplementary Fig. S1c, d). Integrated scRNA-seq and flow cytometry analysis revealed that the cardiac-infiltrating PD1^+^ T cells were derived primarily from the γδ T-cell-related subset (Fig. 1d, e). Flow cytometric analysis further validated the significant post-MI expansion of PD1^+^CD3^+^ T cells in mouse heart tissue, which peaked at 7 days. Compared with those in the sham group, both PD1^+^CD4^+^ and PD1^+^CD8^+^ T-cell subsets were robustly induced after MI and reached maximal levels at 7 days post-MI (Fig. 1f; Supplementary Fig. S2a). Immunofluorescence confirmed the marked induction of PD1^+^ T cells, which peaked at 7 days post-MI (Supplementary Fig. S2b, c). Together, these results highlight dynamic alterations in the PD1^+^ T-cell population, indicating their involvement in MI pathogenesis.

To further describe PD1^+^ T cells under ischemic conditions, peripheral blood samples were obtained from healthy donors and patients with coronary atherosclerosis (AS), including patients with coronary heart disease (CHD) and acute coronary syndrome (ACS). PD1^+^ T-cell frequencies were quantified by flow cytometry. The baseline characteristics of all the participants are provided in Table 1. The frequency of PD1^+^ T cells was greater in AS patients than in healthy controls, peaking in those with ACS (Fig. 1g; Supplementary Fig. S3a). Moreover, mass cytometry of peripheral blood from MI patients at 3 days after onset revealed a marked increase in PD1 expression in CD3^+^ T cells compared with that at admission (Supplementary Fig. S3b). Infarct-area fibrosis was significantly elevated in myocardial infarction patients compared to healthy hearts (Supplementary Fig. S3c, d). In addition, both PD1^+^CD4^+^ T cells and PD1^+^CD8^+^ T cells were significantly enriched in the cardiac tissue of MI patients, exceeding their corresponding frequencies in the peripheral blood (Supplementary Fig. S3e–h). Collectively, these findings confirm that the PD1^+^ T-cell population is induced in MI patients.

**Table 1:** Demographic and clinical characteristics of the patients at baseline.

Characteristic	healthy donor, *n* = 14	AS, *n* = 62	CHD, *n* = 116	STEMI, *n* = 90
Age, Mean ± SD	60.20 ± 13	64.34 ± 10.53	63.52 ± 11.72	63.37 ± 13.53
Sex, *n* (%):				
Female	9 (64.3%)	25 (40.3%)	34 (29.3%)	18 (20.0%)
Male	5 (35.7%)	37 (59.7%)	82 (70.7%)	72 (80.0%)
Diabetes, *n* (%):				
No	13 (92.9%)	52 (83.9%)	80 (69.0%)	65 (72.2%)
Yes	1 (7.1%)	10 (16.1%)	36 (31.0%)	25 (27.8%)
Hypertension, *n* (%):				
No	11 (78.6%)	29 (46.8%)	48 (41.4%)	37 (41.1%)
Yes	3 (21.4%)	33 (53.2%)	68 (58.6%)	53 (58.9%)
Hyperlipidemia, *n* (%):				
No	14 (100.0%)	58 (93.5%)	105 (90.5%)	41 (45.6%)
Yes	0 (0.0%)	4 (6.5%)	11 (9.5%)	49 (54.4%)
Dual antiplatelet therapy, *n* (%):				
No	6 (42.9%)	2 (3.2%)	1 (0.9%)	10 (11.1%)
Yes	8 (57.1%)	60 (96.8%)	115 (99.1%)	80 (88.9%)
Beta-blocker, *n* (%):				
No	9 (64.3%)	44 (71.0%)	49 (42.2%)	22 (24.4%)
Yes	5 (35.7%)	18 (29.0%)	67 (57.8%)	68 (75.6%)
Mineralocorticoid-receptor antagonist, *n* (%):				
No	12 (85.7%)	57 (91.9%)	103 (88.8%)	71 (78.9%)
Yes	2 (14.3%)	5 (8.1%)	13 (11.2%)	19 (21.1%)
Diuretic, *n* (%):				
No	12 (85.7%)	54 (87.1%)	90 (77.6%)	60 (66.7%)
Yes	2 (14.3%)	8 (12.9%)	26 (22.4%)	30 (33.3%)
Statin, *n* (%):				
No	4 (28.6%)	5 (8.1%)	1 (0.9%)	2 (2.2%)
Yes	10 (71.4%)	57 (91.9%)	115 (99.1%)	88 (97.8%)
Angiotensin-converting enzyme (ACE) inhibitor or Angiotensin II receptor blocker (ARB), *n* (%):				
No	12 (85.7%)	34 (54.8%)	48 (41.4%)	35 (38.9%)
Yes	2 (14.3%)	28 (45.2%)	68 (58.6%)	55 (61.1%)
Heart rate, beats/min	67.50 ± 9.55	76.58 ± 14.89	77.44 ± 12.29	80.13 ± 13.81
Systolic blood pressure, mmHg	124.71 ± 14.78	130.45 ± 16.98	134.56 ± 23.78	125.51 ± 16.68
Diastolic blood pressure, mmHg	70.79 ± 12.17	77.44 ± 12.58	77.23 ± 14.88	75.34 ± 11.23
Body mass index	22.83 ± 2.70	25.17 ± 3.15	24.58 ± 3.34	24.13 ± 3.01
Serum creatinine level, μmol/L	67.23 ± 16.21	72.52 ± 15.70	87.72 ± 64.87	101.01 ± 99.39
PD1^+^ T-cell ratio, Mean ± SD				
Total	18.88% ± 4.13%	21.78% ± 7.67%	21.81% ± 7.87%	37.6% ± 12.26%
Female	14.48% ± 4.03%	20.50% ± 7.95%	22.91% ± 14.25%	36.21% ± 14.25%
Male	19.60% ± 4.68%	22.54% ± 7.27%	21.36% ± 7.37%	37.95% ± 11.79%

### PD1^+^ T cells play an immune-activation role in infarcted hearts

To further characterize the molecular features of PD1^+^ T cells in MI, PD1^+^ and PD1^−^ T cells were sorted from WT mouse hearts post-MI for RNA sequencing (Supplementary Fig. S4a, b). PD1^+^ T cells displayed elevated expression of T-cell activation-related genes (*Cd44, Cd69, Nfκb, Cd226, Tnfrsf4 and Icos*), while the expression of exhaustion markers, including TOX and TIGIT, and immune checkpoint molecule CTLA-4 was slightly increased in PD1^+^ T cells compared with PD1^−^ T cells, and the expression of CTLA-4 was relatively low. In contrast, no significant differences were observed in the expression of PD-L2 in infarcted mouse hearts between these two groups (Supplementary Fig. S4c). Furthermore, cytometry showed significantly increased levels of activation markers and secreted proteins (Gzma, IFN-γ, and IL17a) in PD1^+^ T cells relative to PD1^−^ T cells (Fig. 1h, i). We next analyzed PD1^+^ T cells isolated from a Lewis lung carcinoma mouse model to compare their transcriptional profiles with those of cardiac PD1^+^ T cells. Cardiac PD1^+^ T cells displayed reduced expression of exhaustion markers *(Lag3* and *Tim3*) but elevated expression of activation markers (*Icos* and *Cd44*) relative to their counterparts in tumor tissues (Fig. 1j; Supplementary Fig. S4d). These results corroborate the RNA sequencing findings. Collectively, our data indicate that PD1^+^ T cells adopt an activated phenotype in the infarcted heart, suggesting a potentially distinct functional role during MI.

### PD1 deficiency improves cardiac fibrosis post-MI

**Fig. 2:**
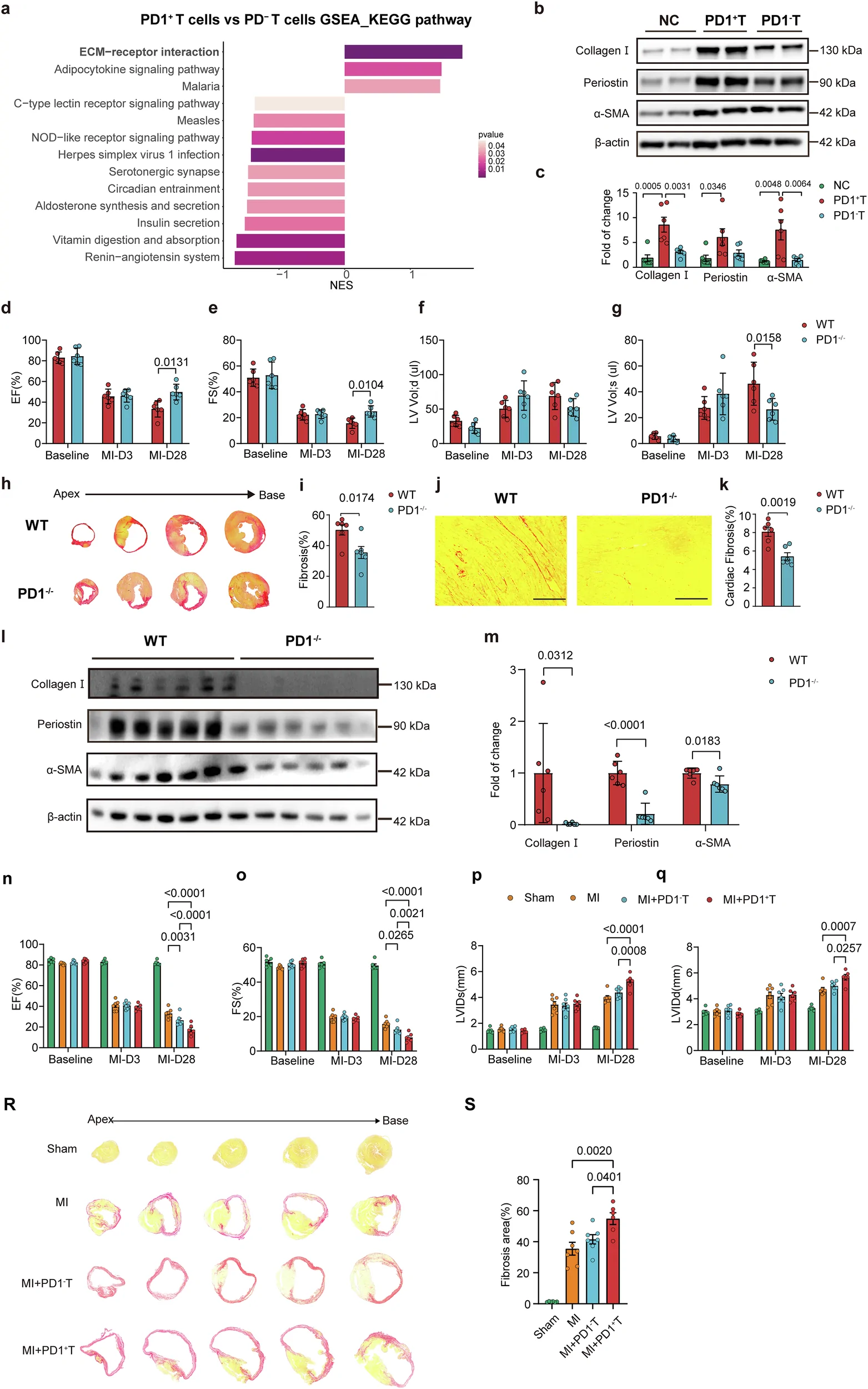
Loss of PD1 expression alleviates cardiac fibrosis accompanied by enhanced cardiac function. **a** GSEA of KEGG signaling pathways in PD1^+^ T cells vs PD1^−^ T cells. **b**, **c** Western blot analysis of ECM proteins, including collagen I, periostin, and α-SMA, in fibroblasts co-cultured with PD1^+^ T cells and PD1^−^ T cells, which were isolated from the mouse heart. Quantitative bar graphs of fibrotic proteins are displayed in **c**; *n* = 6 in each group. **d**–**g** Analyses of left ventricular fractional shortening (LVFS), left ventricular ejection fraction (LVEF), systolic volume (LVSV), and diastolic volume (LVDV) in the WT group and *PD1*^−/−^ group before MI and at 3 days and 28 days after MI; *n* = 6 mice per group. **h** Representative images of Sirius red staining showing the degree of fibrosis in the hearts of WT mice compared with those of *PD1*^−/−^ mice after MI injury. **i** Quantification of scar size in the WT group compared with the *PD1*^−/−^ group after MI injury; *n* = 6 mice per group. **j**, **k** Picrosirius red staining showing different degrees of fibrosis in the remote area of MI hearts from the WT and *PD1*^−/−^ groups, respectively. The fibrosis data are summarized in **k**; *n* = 6 mice per group. Scale bar, 100 μm. **l**, **m** Immunoblots for ECM proteins, including collagen I, periostin, and α-SMA, from WT and *PD1*^−/−^ heart tissues 28 days post-MI. The results of the quantitative analyses are plotted in **m**; *n* = 6 mice per group. **n**–**q** Quantitative analyses of cardiac function parameters in the 4 groups (sham, MI, MI + PD1^−^ T, and MI + PD1^+^ T); *n* = 6–7 in each group. **n** EF; **o** FS; **p** left ventricular internal dimension in systole (LDIDs); **q** left ventricular internal dimension in diastole (LVIDd). **r**, **s** Representative images of Sirius red-stained heart sections from each experimental group. Summary of scar size data is shown in **s**. Two-group comparisons were performed using an unpaired two-tailed Student’s *t*-test. Multiple group comparisons were performed using one-way ANOVA with Tukey’s post hoc test.

To further elucidate the functional role of PD1^+^ T cells post-MI, gene set enrichment analysis (GSEA) of Kyoto Encyclopedia of Genes and Genomes (KEGG) pathways was performed using bulk RNA data from PD1^+^ T and PD1^−^ T cells. PD1^+^ T cells were significantly enriched in the extracellular matrix (ECM)‒receptor interaction pathway (Fig. 2a; Supplementary Fig. S4e), which is a key mediator of pathological fibrosis. To explore the involvement of PD1^+^ T cells in cardiac fibrosis, isolated PD1^+^ T cells and PD1^−^ T cells were individually co-cultured with cardiac fibroblasts. Co-culture with PD1^+^ T cells markedly upregulated fibrosis markers (collagen I, periostin and α-SMA) in cardiac fibroblasts relative to PD1^−^ T cells (Fig. 2b, c), suggesting that PD1^+^ T cells drive fibroblast activation and collagen production. To confirm the functional importance of PD1 in the MI model, global PD1 knockout (*PD1*^*−/−*^) mice were generated, and MI was induced via left anterior descending artery (LAD) ligation in both *PD1*^*−/−*^ and wild-type (WT) mice to evaluate cardiac performance (Supplementary Fig. S5a). Echocardiography demonstrated that compared with WT controls, *PD1*^−/−^ hearts presented markedly improved ejection fraction (EF) and fractional shortening (FS), along with reduced end-diastolic and end-systolic volumes on Day 28 after MI, indicating attenuated ventricular dilation (Fig. 2d–g; Supplementary Fig. S5b). Sirius red staining revealed markedly reduced fibrotic areas in *PD1*^−/−^ hearts relative to WT controls following MI (Fig. 2h–i). Moreover, compared with WT hearts, *PD1*^−/−^ hearts exhibited a marked reduction in remote-zone fibrosis following MI (Fig. 2j, k). The expression of fibrotic markers (α-SMA, collagen I, and periostin) was significantly reduced in *PD1*^−/−^ hearts relative to their WT counterparts following MI (Fig. 2l, m). To confirm that the profibrotic effect was derived from PD1^+^ T cells, we performed adoptive transfer experiments using PD1^−^ T cells as an appropriate control. The transfer of PD1^+^ T cells into mice after MI significantly worsened cardiac function compared with that in the PD1^−^ T group (Fig. 2n–q; Supplementary Fig. S5c) and markedly increased fibrotic deposition, as assessed by Sirius red staining (Fig. 2r, s). Collectively, these results indicate that PD1 deficiency confers protection against post-infarction fibrosis and preserves cardiac performance, whereas PD1^+^ T cells independently drive fibrotic remodeling and functional decline. Wheat germ agglutinin (WGA) staining confirmed a further increase in cardiomyocyte size in the PD1^+^ T-cell transfusion group after MI compared with the MI and PD1^−^ T-cell groups (Supplementary Fig. S5d, e). The survival rate did not significantly differ between these groups (Supplementary Fig. S5f). Collectively, these results demonstrate that PD1 deficiency protects against post-MI fibrosis and preserves cardiac performance, whereas PD1^+^ T cells independently drive fibrotic remodeling and functional deterioration.

### PD1^+^ T cells induce fibroblast activation independent of PD-L1

**Fig. 3:**
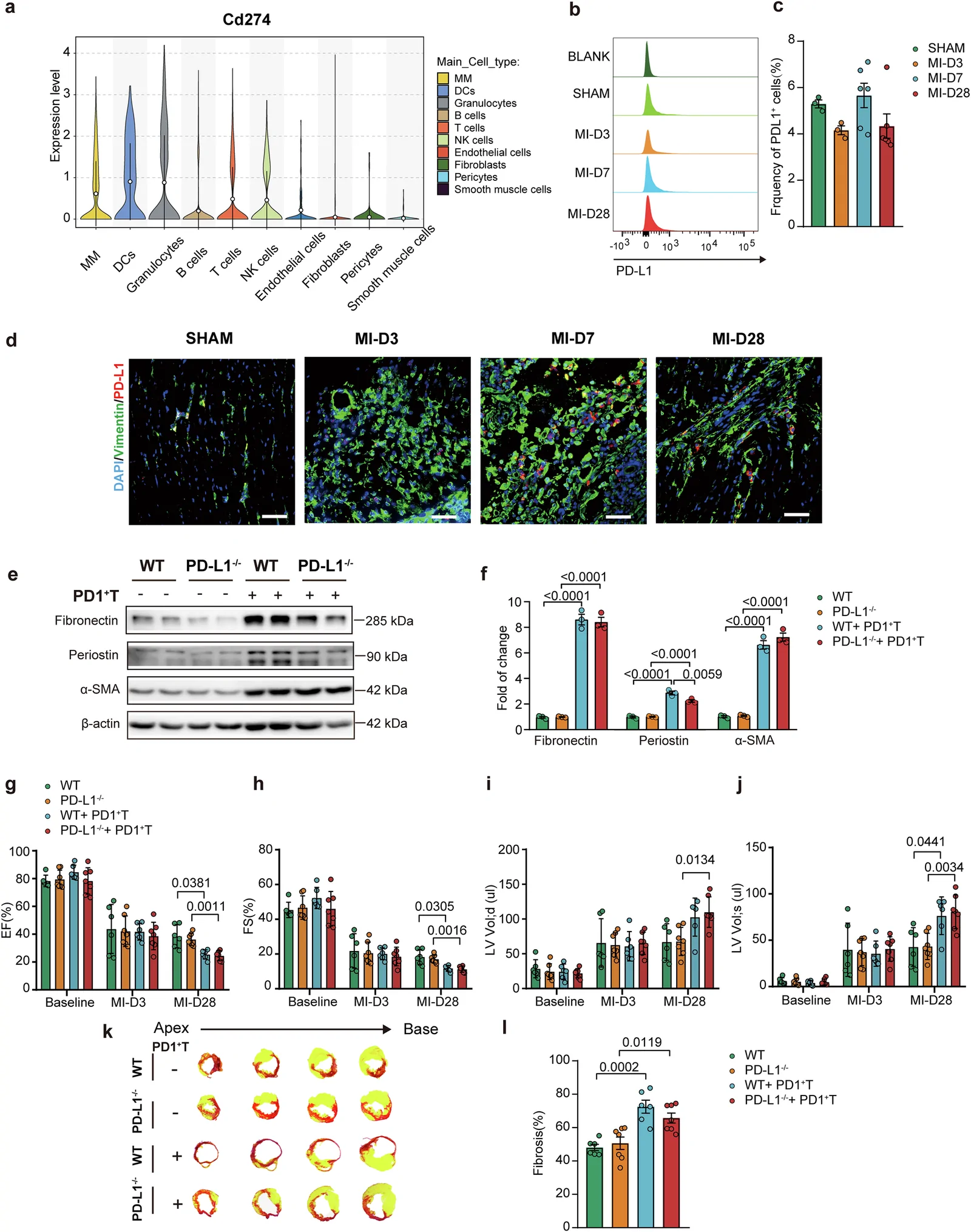
PD1^+^ T cells induce fibroblast activation independent of the PD-L1 pathway. **a** Violin plot of *Cd274* expression in different types of non-myocytes in hearts subjected to MI surgery. **b**, **c** Flow cytometry showing the frequency of PD-L1 expression in isolated cardiac fibroblasts from sham, 3-, 7- and 28-day post-MI hearts; *n* = 3–6 in each group. **d** Immunostaining image showing the location of PD-L1 (red) and vimentin (green) in the infarcted heart on Day 3, 7, 28 after MI. Scale bar, 100 μm. **e**, **f** Expression of extracellular matrix proteins, including extra domain A (EDA)-fibronectin, periostin, and α-SMA, in fibroblasts harvested from WT and *PD-L1*^−/−^ hearts and co-cultured with PD1^+^ T cells; *n* = 3 in each group as indicated. The summary data are plotted in **f**. **g**–**j** LVEF, LVFS, LVSV and LVDV measured by 2-dimensional echocardiography in each experimental group, including WT, *PD-L1*^−/−^, WT with PD1^+^ T-cell infusion and *PD-L1*^−/−^ with PD1^+^ T-cell infusion at 3 days and 28 days following MI; *n* = 6 in the WT group, *n* = 7 in the *PD-L1*^−/−^ group, *n* = 6 in the WT + PD1^+^ T-cell group and *n* = 7 in the *PD-L1*^−/−^ + PD1^+^ T-cell group. **k** Representative images of picrosirius red-stained heart sections from each experimental group. **l** Summary of scar size data; *n* = 6 in the WT group, *n* = 7 in the *PD-L1*^−/−^ group, *n* = 6 in the WT + PD1^+^ T-cell group, and *n* = 7 in the *PD-L1*^−/−^ + PD1^+^ T-cell group. Multiple group comparisons were performed using one-way ANOVA with Tukey’s post hoc test.

As shown above, PD1^+^ T cells in post-MI hearts displayed an active state rather than an exhausted state, which was concomitant with increased fibroblast activation and increased collagen synthesis. Engagement of PD1 with PD-L1 suppresses T-cell activity, leading to diminished inflammatory cytokine production and enhanced T-cell apoptosis^10^. Accordingly, we investigated whether PD1^+^ T-cell-mediated fibrosis following MI is dependent on PD1/PD-L1 signaling. Initial analysis of the scRNA-seq dataset revealed that PD-L1 (encoded by *Cd274*) expression was relatively low in fibroblasts compared with that in other cell populations (Fig. 3a). Notably, PD-L1 expression on fibroblasts was not significantly altered in either the in vivo MI model or in vitro co-culture with PD1^+^ T cells, as evidenced by flow cytometry (Fig. 3b, c; Supplementary Fig. S6a, b) and immunostaining analysis (Fig. 3d). Furthermore, PD-L1 knockout (*PD-L1*^−/−^) mice were generated (Supplementary Fig. S6c). Co-culture of PD1^+^ T cells with primary fibroblasts from either WT or *PD-L1*^−/−^ mice resulted in a comparable increase in collagen secretion in both groups (Fig. 3e, f). To verify whether PD1^+^ T cells modulate cardiac function and fibrosis independent of PD-L1 in vivo, an MI model was generated in WT and *PD-L1*^−/−^ mice, both of which received PD1^+^ T-cell infusions. PD1^+^ T-cell infusion similarly exacerbated cardiac dysfunction in both WT and *PD-L1*^−/−^ mice following MI compared with their respective controls without PD1^+^ T-cell infusion. Although adoptive transfer of PD1^+^ T cells significantly impaired cardiac function in both genotypes, no significant difference was observed between WT and *PD-L1*^−/−^ MI mice in the presence or absence of PD1^+^ T-cell infusion (Fig. 3g–j; Supplementary Fig. S6d–g). Sirius red staining indicated comparable increases in cardiac fibrosis in WT and *PD-L1*^−/−^ mice following PD1^+^ T-cell infusion, indicating that fibrosis is driven independently of the PD1/PD-L1 signaling pathway (Fig. 3k, l). Collectively, these results indicate that PD1^+^ T-cell-mediated fibrosis after MI occurs independent of PD-L1 ligand–receptor interactions.

### PD1^+^ T cells promote fibrosis through the CXCL9/CXCR3 axis

**Fig. 4:**
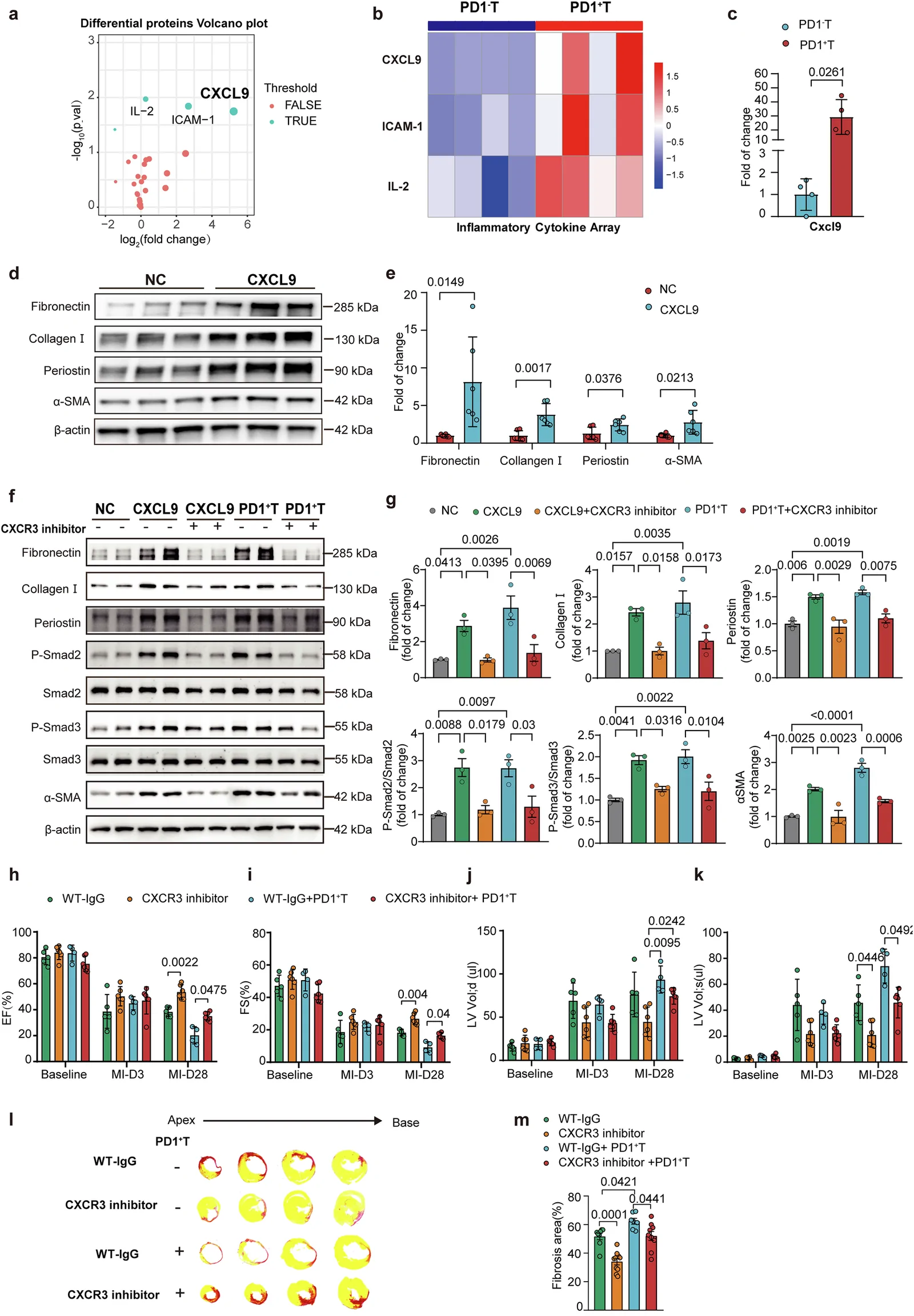
PD1^+^ T cells induce cardiac fibroblast activation through the CXCL9/CXCR3 axis. **a** Volcano plot of the differentially abundant cytokines between PD1^+^ T cells and PD1^−^ T cells according to the *P*-value and fold change. **b** Heatmap of significantly differentially expressed inflammatory cytokines (CXCL9, ICAM-1, and IL-2) between PD1^+^ T cells and PD1^−^ T cells. **c** Relative quantification of CXCL9 expression in the cytokine array between PD1^+^ T cells and PD1^−^ T cells; *n* = 4 in each group. **d**, **e** Western blot showing the expression of fibrotic proteins in neonatal cardiac fibroblasts treated with recombinant cytokine CXCL9 or the control. Summary data are plotted in **e**. **f**, **g** Representative western blot analyses showing the expression of fibrosis-associated proteins (fibronectin, collagen I, α-SMA and periostin) and TGF-β/Smad signaling molecules in mouse cardiac fibroblasts after 48 h of treatment. The summary data are plotted in **g**. **h**–**k** In the mouse model, CXCR3 inhibitors were given to WT mice to block the CXCL9/CXCR3 pathway (300 µg/mouse every 3 days until sacrifice), and an isotype was used as the control. The LVEF, LVFS, LVSV, and LVDV were measured by 2-dimensional echocardiography in each experimental group, including the WT mice treated with isotype control (IgG), CXCR3 inhibitor, IgG with PD1^+^ T-cell infusion, and CXCR3 inhibitor with PD1^+^ T-cell infusion groups on Day 3 and Day 28 following MI (*n* = 4–6 in each group). **l**, **m** Representative images of picrosirius red staining of sequential heart sections from each experimental group. Summary data of scar size are plotted in **m**; *n* = 7–10 in each group.

To elucidate the mechanisms driving the pro-fibrotic activity of PD1^+^ T cells, we analyzed the cytokine secretomes of PD1^+^ T cells and PD1^−^ T cells sorted from post-MI mouse hearts for inflammatory cytokine profiling. Both types of cells sorted from post-MI mouse hearts were analyzed for inflammatory cytokines. The levels of three cytokines (CXCL9, ICAM1, and IL-2) differed significantly between these two cell types, with CXCL9 being the top-ranking cytokine according to the fold change index (Fig. 4a–c). Consistent with previous reports showing that CXCL9 activates fibroblasts via CXCR3^11^, exogenous CXCL9 stimulation increased the expression of pro-fibrotic markers (fibronectin, Collagen I, periostin and α-SMA) in cardiac fibroblasts and induced a myofibroblast-like phenotype (Fig. 4d, e; Supplementary Fig. S7a). Collectively, these findings suggest that PD1^+^ T-cell-derived inflammatory cytokines may promote fibroblast activation via the CXCL9/CXCR3 axis.

To assess whether blocking the CXCL9/CXCR3 axis could suppress PD1^+^ T-cell-induced fibrosis, PD1^+^ T cells were co-cultured with fibroblasts in vitro with a CXCL9/CXCR3 axis inhibitor. Inhibition of CXCR3 significantly reduced fibroblast activation driven by PD1^+^ T cells (Fig. 4f, g). CXCR3 inhibition significantly reduced CXCL9-induced pro-fibrotic protein expression and phosphorylation of SMAD2/3. These findings indicate that disrupting the CXCL9/CXCR3 interaction in fibroblasts effectively mitigates fibrosis and downstream profibrotic signaling (Fig. 4f, g). After confirming the dependence of PD1^+^ T-cell-induced fibrosis on the CXCL9/CXCR3 axis in vitro, we demonstrated that CXCR3 inhibition preserved cardiac function following PD1^+^ T-cell administration in vivo (Supplementary Fig. S7b). At 28 days post-MI, echocardiography revealed that CXCR3 inhibition significantly mitigated PD1^+^ T-cell-induced cardiac dysfunction, as reflected by increased EF% and FS%, decreased end-systolic and end-diastolic volumes (Fig. 4h–k; Supplementary Fig. S7c), and reduced fibrotic remodeling (Fig. 4l, m). To further determine whether PD1^+^ T cells exert their effects through the CXCL9/CXCR3 axis, we performed combination blockade experiments with both PD1 and CXCR3 inhibitors in vitro. Notably, compared with PD1 blockade alone, combined inhibition did not result in a further reduction in the expression of fibrosis-related markers (Supplementary Fig. S7e, f), suggesting that the pro-fibrotic effects of PD1^+^ T cells are largely mediated through the CXCL9/CXCR3 axis. To elucidate the upstream transcriptional regulation of CXCL9, TRRUST database analysis was performed and revealed three candidate transcription factors: IKBKB, IRF4, and STAT1. Among them, IRF4 was uniquely and significantly upregulated in PD1^+^ cells, as shown by bulk RNA-seq and scRNA-seq on Day 7 post-MI (Supplementary Fig. S7d, g). Consistent with these findings, IRF4 expression was elevated in PD1^+^ T cells from infarcted hearts compared with sham hearts (Supplementary Fig. S7h). qPCR analysis confirmed robust upregulation of IRF4 expression in PD1^+^ T cells compared with PD1^−^ T cells (Supplementary Fig. S7i), identifying IRF4 as a likely upstream regulator of CXCL9. Collectively, these results revealed that PD1^+^ T-cell-driven post-MI fibrosis occurs via activation of the CXCL9/CXCR3 axis in fibroblasts.

### Anti-PD1 therapy mitigates fibrosis post-MI in mice

**Fig. 5:**
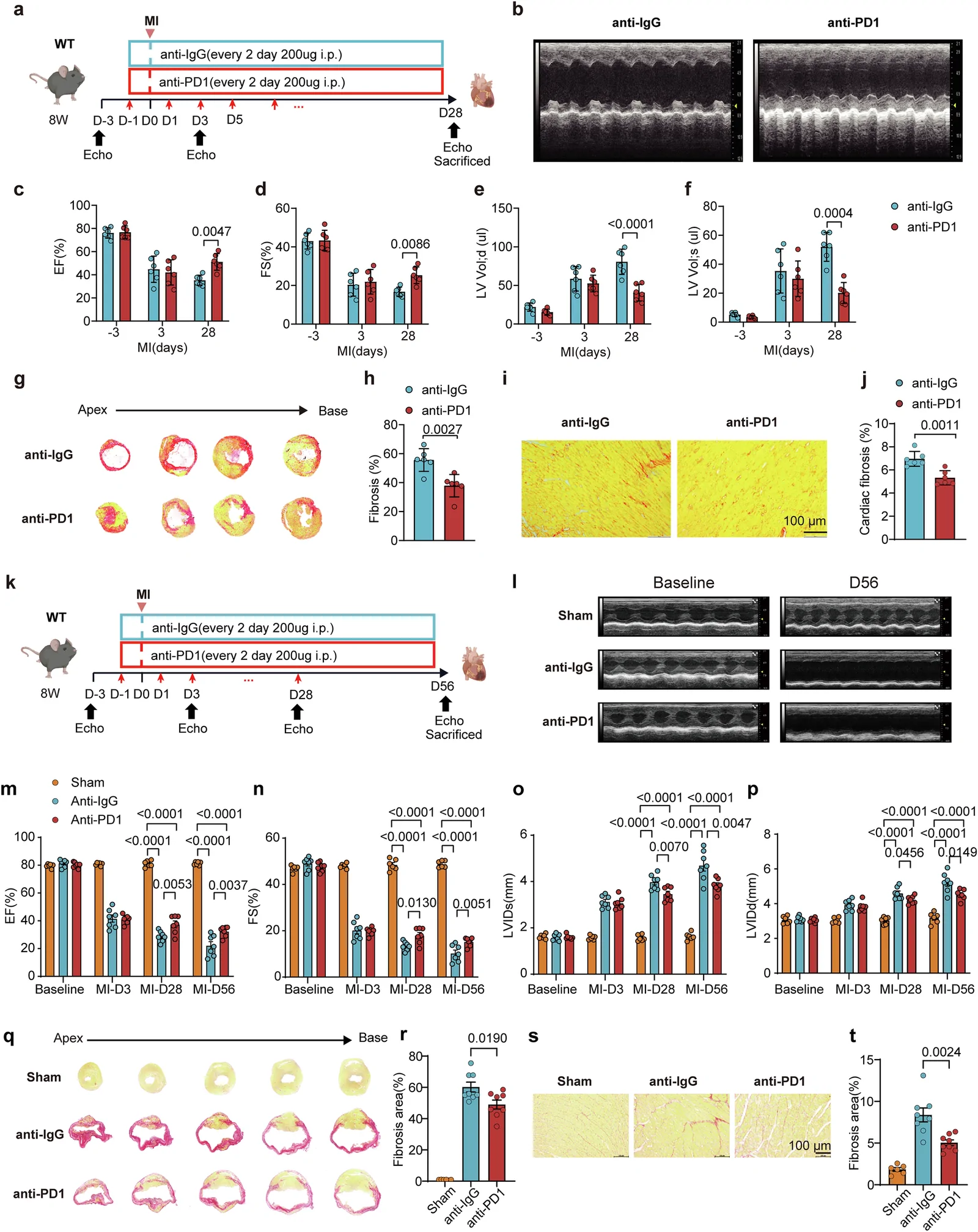
PD1 blockade alleviates cardiac fibrosis and improves cardiac function. **a** Study design for WT mice treated with an anti-PD1 inhibitor or anti-IgG antibody post-MI. **b** Representative M-mode images of WT mice after anti-PD1 inhibitor or IgG administration post-MI. **c**–**f** Evaluation of cardiac function by echocardiography revealed the LVEF, LVFS, LVSV, and LVDV in mice subjected to PD1 antibody injection before MI and every 2 days at 200 µg/mouse to 28 days, with isotype injection used as the control group; *n* = 6 mice per group. **g**, **h** Representative images of Sirius red-stained heart sections from each experimental group. Summary data of scar size are plotted in **h**; *n* = 6 in each group. **i** Representative images of interstitial fibrosis in the remote area after MI in each group. **j** Fibrosis quantification results are plotted as a percentage of the fibrotic area (mean with SEM); *n* = 6 per group. **k** Study design for WT mice receiving anti-PD1 inhibitor or anti-IgG injection post-MI. **l** Representative M-mode images of WT mice after anti-PD1 inhibitor or IgG injection post-MI. **m**–**p** Cardiac function evaluated by echocardiography revealed the LVEF, LVFS, LVIDs, and LVIDd in mice subjected to PD1 antibody injection before MI and every 2 days with 200 µg/mouse for up to 56 days, with the control group receiving an isotype injection; *n* = 6–7 mice per group. **q**, **r** Representative images of picrosirius red-stained heart sections from each experimental group. Summary data of scar size are plotted in **r**; *n* = 6–8 in each group. **s** Representative images of interstitial fibrosis in the remote area after MI in each group. **t** Fibrosis quantification results are plotted as a percentage of the fibrotic area; *n* = 6–8 per group. Scale bar, 100 μm. Two-group comparisons were performed using an unpaired two-tailed Student’s *t*-test. Multiple group comparisons were performed using one-way ANOVA with Tukey’s post hoc test.

Given that PD1^+^ T cells drive fibrotic remodeling following MI, we next explored the therapeutic potential of targeting PD1 to mitigate cardiac dysfunction. The mice received an intraperitoneal injection of a PD1 inhibitor every two days after MI (Fig. 5a). Notably, the PD1 inhibitor did not increase mortality throughout the 28-day observation period, indicating favorable tolerability in vivo. Functionally, compared with placebo treatment, PD1 inhibitor treatment significantly preserved cardiac performance, as evidenced by the improved EF and FS, together with reduced left ventricular end-diastolic and end-systolic volumes (Fig. 5b–f). Histological assessment further revealed that the PD1 inhibitor attenuated fibrotic remodeling, reducing both infarct length and interstitial fibrosis in the remote myocardium (Fig. 5g–j). Collectively, these findings highlight pharmacological blockade of PD1 as a promising therapeutic strategy to alleviate post-MI fibrosis and preserve cardiac function.

To evaluate the long-term therapeutic potential of anti-PD1 treatment for MI, we administered an anti-PD1 antibody intraperitoneally every two days for 56 days (Fig. 5k). Remarkably, prolonged anti-PD1 therapy continued to confer a cardioprotective effect, as evidenced by preserved cardiac function and reduced left chamber volume (Fig. 5l–p). Consistent with these findings, Sirius red staining revealed a significantly decreased fibrotic length and reduced fibrosis area in both the infarct zone and remote zone in anti-PD1-treated mice compared with IgG control mice (Fig. 5q–t). To further examine the immunomodulatory effects of PD1 inhibition, we analyzed cardiac-infiltrating T-cell subsets, including IFN-γ^+^CD4^+^ type 1 T helper (Th1) cells, IL-4^+^CD4^+^ Th2 cells, IL-17^+^CD4^+^ Th17 cells, CD25^+^FOXP3^+^CD4^+^ regulatory T cells (Tregs), and CD8^+^ T cells from CD45^+^CD3^+^ populations, on Day 7 post-MI (Supplementary Fig. S8a). However, it modestly decreased the Th1 cell frequency while increasing the Treg abundance (Supplementary Fig. S8b–f), indicating a shift toward a more anti-inflammatory immune environment. In addition, to further evaluate the protective effect of the PD1 inhibitor on cardiomyocytes, we conducted TUNEL analysis on cardiomyocytes from both groups. No significant difference was detected between the two groups (Supplementary Fig. S8g, h); the blood pressure also did not differ after MI between the anti-PD1 and control groups (Supplementary Fig. S8i, j). WGA staining confirmed a further decrease in cardiomyocyte size in PD1 inhibitor-treated mice after MI (Supplementary Fig. S8k, l), whereas no significant difference in survival rates after MI was observed between the anti-PD1 and control groups (Supplementary Fig. S8m). Collectively, these findings demonstrate that anti-PD1 therapy effectively mitigates cardiac fibrosis and preserves cardiac function post-MI, providing both short- and long-term therapeutic benefits, which supports its translational potential for post-MI cardiac remodeling.

### Anti-PD1 therapy ameliorates cardiac fibrosis post-MI in non-human primates

**Fig. 6:**
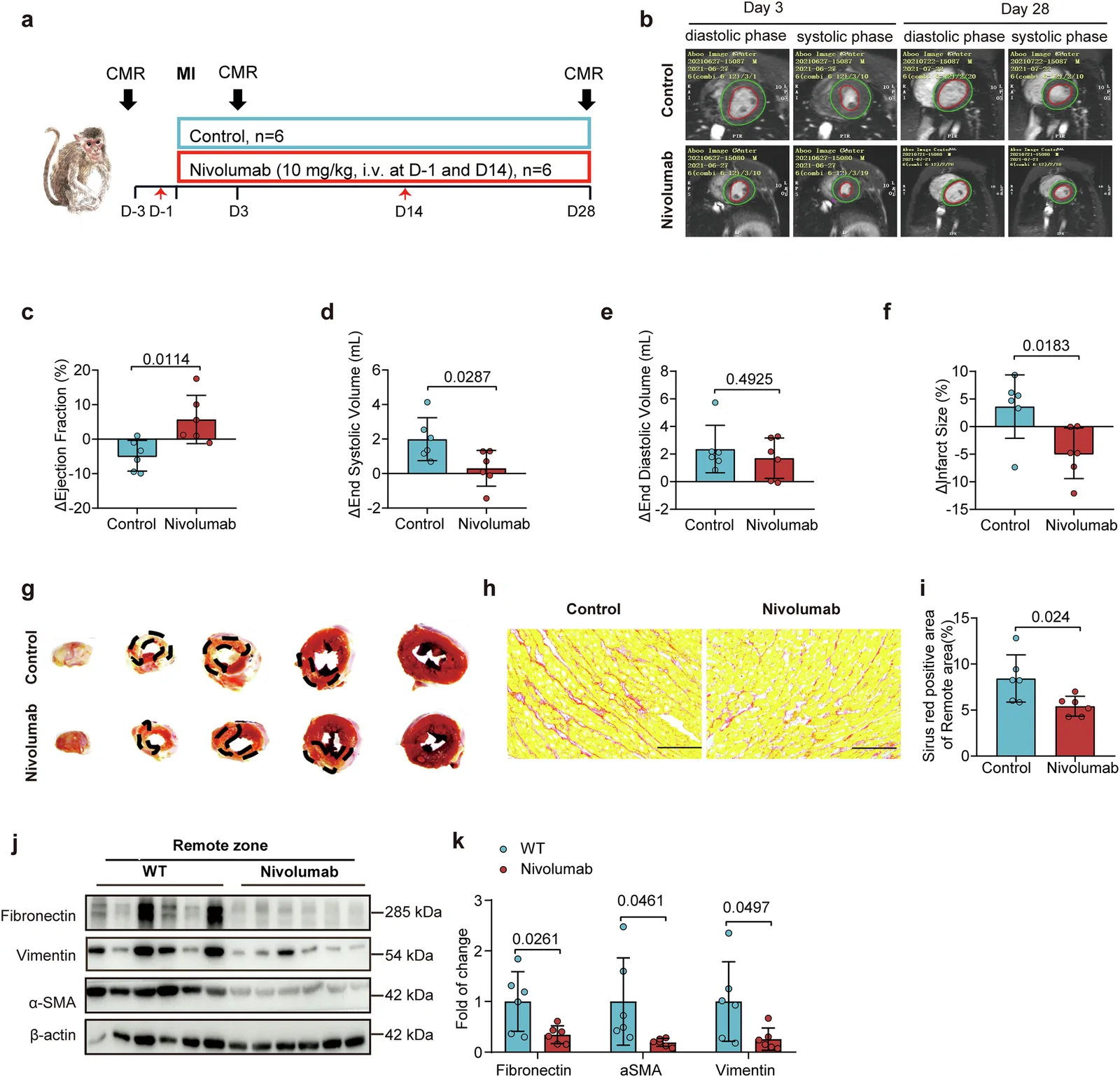
Nivolumab moderates cardiac fibrosis in infarcted non-human primate hearts. **a**, **b** MI was surgically induced in cynomolgus monkeys, followed by treatment with saline or nivolumab (10 mg/kg, two doses on the day before surgery and 14 days after MI). **c**–**e** Cardiac MRI assessments were conducted 3 days and 1 month after MI and treatment and were used to calculate the change (Δ) in the LVEF, ESV and EDV between the two time points (saline group, *n* = 6; nivolumab group, *n* = 6). **f** Summary of infarct size data determined by cardiac MRI assessments in the control group and nivolumab group. **g** Heart tissue sections from apex to base were collected at 1 month. **h**, **i** Representative images of interstitial fibrosis in non-infarcted areas in the heart from each experimental group. Summary data on fibrosis percentage are plotted in **i**; *n* = 6 in each group. Scale bar, 200 μm. **j**, **k** Expression of fibrosis-related proteins in heart tissues harvested from monkey hearts in each group; *n* = 6 in each group as indicated. Two-group comparisons were performed using an unpaired two-tailed Student’s *t*-test.

To investigate the translational potential of the PD1 inhibitor as a therapeutic strategy against post-MI cardiac remodeling, cynomolgus monkeys undergoing MI were treated with either a vehicle or nivolumab, a clinically approved PD1 inhibitor. Nivolumab or the vehicle was administered intravenously one day before MI induction and again on Day 14 after MI. Cardiac magnetic resonance imaging (MRI) was performed at 3 and 28 days post-MI to assess cardiac function across treatment groups (Fig. 6a). Flow cytometry revealed a marked reduction in the number of circulating PD1^+^ T cells in nivolumab-treated monkeys (Supplementary Fig. S9a). From Day 3 to Day 28 after MI, nivolumab treatment resulted in a positive change in EF (ΔEF), in contrast to the negative ΔEF observed in vehicle-treated controls (Fig. 6b, c). Similarly, the end-systolic volume (ΔESV) significantly decreased in nivolumab-treated hearts, whereas the end-diastolic volume (ΔEDV) showed a similar decreasing trend without statistical significance (Fig. 6d, e). The reduction in infarct size from Day 3 to Day 28 was significantly greater in the nivolumab-treated monkeys than in the controls (Fig. 6f, g). Sirius red staining further revealed reduced fibrosis in the remote myocardium on Day 28 post-MI in the nivolumab-treated group (Fig. 6h, i). In parallel, western blot analysis revealed decreased expression of fibrosis markers, including fibronectin, vimentin, and α-SMA, in the nivolumab-treated hearts compared with the control hearts (Fig. 6j, k).

To comprehensively assess the safety of nivolumab treatment following MI, we analyzed systemic inflammation as well as hepatic, renal, and thyroid functions in both groups. Cytokine array analysis revealed that nivolumab did not significantly increase the risk of cytokine storm, as indicated by comparable levels of IL-6 and IL-4 between the nivolumab-treated and control groups (Supplementary Fig. S9b). Hepatic function, assessed by measuring serum alanine aminotransferase and alkaline phosphatase levels, did not significantly differ between the groups (Supplementary Fig. S9c, d). Similarly, the levels of renal function markers, including creatinine and blood urea nitrogen (Supplementary Fig. S9e, f), and the levels of thyroid hormones, free triiodothyronine and free thyroxine (Supplementary Fig. S9g, h), remained within normal ranges in both groups. Blood pressure also did not differ after MI between the anti-PD1 and control groups (Supplementary Fig. S9i, j). Collectively, these findings demonstrate that nivolumab administration significantly ameliorated post-MI cardiac fibrosis and improved cardiac function while maintaining favorable safety profiles across major systems in a non-human primate model. These results strongly support the translational potential of nivolumab as a promising therapeutic strategy for patients with MI.

### PD1^+^ T frequency is a potent prognostic biomarker for MI patients

**Fig. 7:**
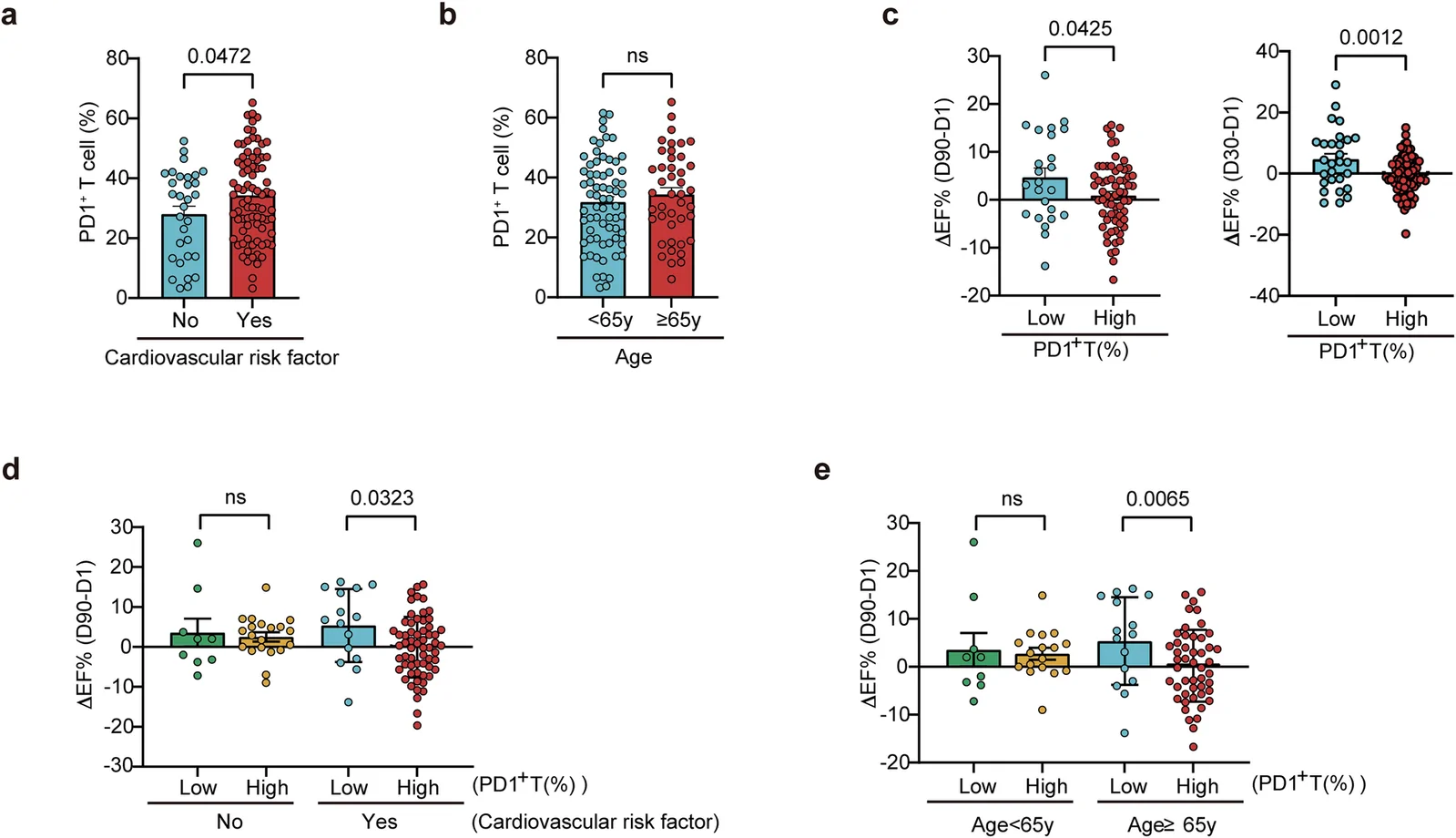
Prognosis of MI patients with different layers of PD1^+^ T cells. **a** Association between cardiovascular risk status and PD1^+^ T-cell proportions. **b** Comparison of PD1^+^ T-cell proportions by age stratification. **c** Echocardiographic analysis revealed that patients with lower PD1^+^ T-cell proportions (< 20%) exhibited greater improvement in EF from baseline to Day 30 and Day 90 post-MI. **d** Functional recovery by PD1^+^ T-cell levels in the low-risk and high-risk cohorts (Day 90 post-MI). **e** Functional recovery by PD1^+^ T-cell levels in different age stratification cohorts (Day 90 post-MI). The proportion of PD1^+^ T cells increased from healthy donors to patients with STEMI, and 20% served as a cut-off value to distinguish coronary artery disease (CAD, including STEMI) patients from healthy controls or AS patients.

To further evaluate the clinical relevance of PD1^+^ T cells in cardiac function, we analyzed peripheral blood and echocardiogram data from patients with STEMI. We observed that individuals with established cardiovascular risk factors (e.g., hypertension, diabetes, and hyperlipidemia) tended to have a higher proportion of circulating PD1^+^ T cells; notably, elderly patients displayed levels comparable to those of their younger counterparts (Fig. 7a, b). Consistent with previous data, the average proportion of PD1^+^ T cells in peripheral blood among CHD and STEMI patients was approximately 20%, whereas that in healthy donors was typically < 20%. Accordingly, a 20% cutoff value was used to evaluate patient prognosis.

At 90 days post-MI, patients with a higher PD1^+^ T ratio (≥ 20%) exhibited significantly impaired cardiac function compared with those with a lower ratio (< 20%) (Fig. 7c), suggesting that post-MI, PD1^+^ T-cell expansion is closely associated with adverse cardiac outcomes. Subgroup analyses further revealed that among the higher-risk groups, elderly patients with elevated PD1^+^ T-cell levels (≥ 20%) had markedly worse cardiac function at the 90-day follow-up (Fig. 7d, e).

Collectively, these clinical data reinforce our experimental observations and demonstrate that increased peripheral PD1^+^ T-cell levels post-MI correlate strongly with poor cardiac remodeling and dysfunction, underscoring the potential of peripheral PD1^+^ T cells as a prognostic biomarker for adverse cardiovascular outcomes.

## Discussion

Cardiac fibrosis and the inflammatory response are integral to heart failure following MI. Fibroblast activation is now recognized as the primary cause of fibrosis. Our study elucidates a previously unrecognized role of PD1^+^ T cells in promoting cardiac fibrosis post-MI. We demonstrated that blocking the activation of PD1^+^ T cells using a PD1 inhibitor in both rodents and non-human primates mitigated its pro-fibrotic effect, suggesting that it is a novel therapeutic target for cardiac fibrosis subsequent to MI.

PD1^+^ T cells have been implicated in inflammatory diseases such as rheumatoid arthritis and COVID-19, where they express cytokines such as TNF-α, IFN-γ, and IL-2^8,9^. Although T cells are known to participate in myocardial injury and repair, the role of PD1^+^ T cells remains unclear. We detected marked post-MI expansion of activated PD1^+^ T cells with high ICOS, CD44, IFN-γ, and IL-17a expression. Unlike the canonical model in tumors, in which PD-L1 binding to PD1 leads to T-cell exhaustion via SHP2 activation^12–14^ — our data suggest a PD-L1-independent role for PD1^+^ T cells in promoting fibrosis. It remains possible that other PD-L1^+^ cardiac cell populations modulate the functional state of PD1^+^ T cells within the cardiac niche. However, compared with other cardiac cell types, cardiac fibroblasts, which are the primary effector cells driving fibrosis in MI, express relatively low levels of PD-L1. Consistent with this, we did not observe evidence of SHP2 phosphorylation in PD1^+^ T cells during co-culture with fibroblasts, further indicating minimal functional PD1/PD-L1 engagement in this context. Moreover, PD1^+^ T cells activated fibroblasts even in the absence of PD-L1, both in vitro and in vivo, suggesting alternative pathways, such as direct cell–cell contact or fibrogenic cytokine secretion.

We further identified the CXCL9/CXCR3 axis as a critical mediator of PD1^+^ T-cell-driven fibrosis. CXCL9, a member of the CXC chemokine family, interacts with the chemokine receptor CXCR3 to mediate its chemotactic function. CXCL9 is involved in Th1-type inflammation and primarily recruits activated B cells, monocytes, CD8^+^ memory T cells, and CD4^+^ Th1 T cells. Previous studies have shown that CXCL9 can promote T-cell recruitment and fibroblast activation. In tumors, CXCL9 enhances PD1 inhibitor efficacy by attracting T cells; in chronic rheumatic heart disease, it facilitates T-cell infiltration at injury sites^15^. In our study, we demonstrated that PD1^+^ T cells exhibited elevated CXCL9 production. Through transcription factor analysis based on both bulk RNA-seq and scRNA-seq datasets on Day 7 post-MI, we found that the upstream regulator IRF4 was significantly upregulated in PD1^+^ T cells compared with PD1^−^ T cells. Previous studies have demonstrated that IRF4 can directly activate the CXCL9 promoter and increase its transcription. Together, these findings suggest that IRF4 is a potential key mediator driving CXCL9 upregulation in PD1^+^ T cells following MI. Notably, we further demonstrated that inhibition of CXCL9/CXCR3 axis effectively suppressed PD1^+^ T-cell-induced fibroblast activation and reduced cardiac fibrosis.

PD1 blockade is well studied in oncological contexts, where it restores cytotoxic T-cell function. Our study extends its utility to cardiac fibrosis, showing that PD1 inhibition via nivolumab reduces fibrosis and improves function in MI models. Interestingly, PD1 inhibition increased the number of Tregs and reduced the number of Th1 cells, indicating an anti-inflammatory shift. Immune checkpoint blockade (ICB) can induce the activation of T cells that target tumor cells, although some patients exhibit non-response or even accelerated progression after treatment. The efficacy of ICB relies on tumor-reactive T cells. Over the past decade, research has increasingly highlighted the role of anti-PD1 in inducing T-cell proliferation and reversing T-cell exhaustion. However, in some inflammatory diseases, anti-PD1 therapy can induce an immunosuppressive environment. In lung tumor models, PD-L1 does not suppress cytotoxic lymphocyte activity^16^. Previous studies have demonstrated that ICB can promote metabolic fitness and increase T-cell infiltration in tumors with low levels of glycolysis^17^. Under low-lactate conditions, CD8^+^ T effector cells function normally, whereas under high-lactate conditions, the proliferation and suppressive function of Tregs increase^18^. Blocking lactate can improve the efficacy of checkpoint therapy^19^. Under high-lactic acid conditions, the mechanism underlying anti-PD1 therapy may preferentially shifts from promotion of CD8^+^ T cells to enhancement of Treg activity, which is consistent with our findings. During MI, a decrease in mitochondrial oxidative phosphorylation and an increase in the glycolytic rate of cardiac myocytes lead to elevated lactate levels^20^. This shift suggests that high lactic acid levels convert PD1 blockade from an immune-activating state to an immunosuppressive state. These findings support the hypothesis that PD1^+^ T cells function independently of the PD-L1 axis.

Our findings emphasize the disease-specific effects of PD1 blockade. In cancer, PD1^+^ T cells are exhausted and cytotoxic, whereas in MI, they are activated and profibrotic — likely derived from peripheral recruitment or local expansion. In our model, PD1 inhibition reduced both the abundance and fibrotic activity of these cells, possibly by disrupting immune–stromal interactions or altering local cytokine signals (e.g., CXCL9). These results suggest that, unlike in cancer, PD1 blockade in MI suppresses maladaptive inflammation and fibrosis rather than reactivating T-cell cytotoxicity. This highlights the importance of the local immune context in shaping the outcomes of PD1-targeted therapy. Notably, this mechanism is distinct from that of conventional antifibrotic agents such as ACE inhibitors or mineralocorticoid receptor antagonists, which primarily act through systemic RAAS inhibition. PD1 blockade offers a complementary approach by targeting immune–fibroblast crosstalk within the heart, largely independent of renin-angiotensin-aldosterone system (RAAS) signaling. Therefore, combining PD1 inhibitors with standard therapies may yield additive or synergistic effects by modulating parallel fibrotic pathways — systemic neurohormonal signaling and local immune-driven remodeling. This strategy may offer a more comprehensive means of mitigating post-MI fibrosis and its clinical consequences.

Despite these promising results, important limitations remain. Our study demonstrated the short-term therapeutic benefit of PD1 inhibition in post-MI cardiac fibrosis, but its long-term safety remains uncertain. Chronic PD1 blockade has been associated with autoimmune complications such as myocarditis, pneumonitis, and colitis — particularly in the oncological setting, where sustained immune activation is common^21–23^. The combination of a PD1 inhibitor with other ICB, particularly CTLA-4 inhibitors, led to the development of myocarditis^24^. In tumors, anti-PD1 therapy primarily increases the ratio of cytotoxic and effector CD8^+^ T cells. In the cancer microenvironment, PD1 expression typically suppresses TCR functions and induces T-cell exhaustion. Activated CD8^+^ T cells are predominantly associated with adverse immune responses^25^. *PD1*^−/−^ mice do not typically exhibit a distinct phenotype under normal conditions. However, when both PD1 and CTLA-4 are knocked out, these mice develop severe myocarditis characterized by massive CD8^+^ T-cell infiltration in the heart^26^. Notably, in our model, PD1 inhibition altered CD4^+^ subsets without expanding CD8^+^ T cells or triggering autoimmune features during the experimental window. However, the lack of long-term follow-up is a key limitation, and further studies are needed to evaluate the efficacy of treatment, immune homeostasis, and immune-related adverse events (irAE) risk.

Translational application of PD1 blockade in MI — especially in elderly or comorbid populations — requires careful risk–benefit assessment. Unlike in cancer, where PD1^+^ T cells are exhausted, those in MI are immunologically active and pathogenic. Our findings suggest that PD1 inhibition suppresses these profibrotic responses without broadly activating immunity. We observed no signs of CD8^+^ T-cell expansion or systemic toxicity, suggesting a more favorable safety profile in acute cardiac injury. Nevertheless, the risk of irAEs — albeit low with PD1 monotherapy in cancer (~0.5% incidence of myocarditis) — must be carefully weighed against therapeutic benefit. Acute inflammation is indispensable for infarct healing; therefore, the optimal timing of PD1 blockade requires careful consideration. Although anti-PD1 treatment initiated around the time of MI improved cardiac remodeling in our study, delayed intervention after the acute inflammatory phase may maximize therapeutic benefit while minimizing interference with early tissue repair. Future studies should systematically evaluate the therapeutic window of PD1-targeted intervention after MI, including delayed administration strategies.

Importantly, early stratification strategies may improve the clinical safety of PD1 inhibitors in MI. In our preliminary human data, elevated levels of circulating PD1^+^ T cells (> 20%) post-MI were associated with metabolic comorbidities and poor cardiac recovery, suggesting that these cells could be potential biomarkers for patient selection. Ongoing efforts to establish MI cohorts within cancer populations, alongside upcoming early-phase clinical trials, aim to identify predictive biomarkers for both efficacy and irAE susceptibility. These studies will integrate immune phenotyping and cardiac imaging to refine patient selection and optimize treatment safety.

In conclusion, our findings revealed a novel role for PD1^+^ T cells in cardiac fibrosis during the post-MI remodeling process and revealed that PD1 inhibition could serve as an effective treatment strategy. Further research is necessary to elucidate more detailed molecular mechanisms underlying the preventive effect of PD1 inhibition under MI conditions, which would facilitate its translation into clinical practice.

## Materials and Methods

### Animal ethics

In accordance with the Guidelines for the Care and Use of Laboratory Animals in China, this study was approved by the Laboratory Animal Ethics Committee of Zhejiang University. C57BL/6 J mice (8–10 weeks old, male) were purchased from Shanghai Slac Laboratory Animal Co., Ltd. The animals were housed in SPF-grade experimental rooms at the Animal Experiment Center of the Second Affiliated Hospital of Zhejiang University School of Medicine under controlled conditions of 25 °C and a 12 h:12 h light/dark cycle and fed standard laboratory chow. C57BL/6 J neonatal mice, which were less than 24 h old, were purchased from the Zhejiang Academy of Medical Sciences. Cynomolgus monkeys (males; 3–5 years old; 5–6 kg of body weight) were obtained from Suzhou Xishan Zhongke Laboratory Animal Co., Ltd., which has been certified by the Association for Assessment and Accreditation of Laboratory Animal Care. All analyses were double-blind and randomized.

### Establishment of a mouse MI model and intramyocardial injection

Eight- to ten-week-old adult C57 mice (weighing 22 g) were used to establish the MI model. The mice were anesthetized with 4% pentobarbital (10 mg/0.8 mL), and a 20 G intravenous catheter (BD) connected to a small animal ventilator through a three-way stopcock was used for intubation. Ventilation was performed with an adjusted tidal volume of 1.0–1.5 mL and a respiratory rate of 95–100 breaths per minute. A thoracotomy was performed, and a small animal chest retractor was used to spread the ribs, fully exposing the heart. The left anterior descending coronary artery was permanently ligated with a 7–0 silk suture approximately 1–2 mm below the left atrial appendage. Successful MI was indicated by whitening of the apex due to loss of blood supply.

Spleens were harvested from mice on Day 7 after MI and mechanically dissociated through a 70 μm cell strainer to obtain single-cell suspensions. Red blood cells were lysed using standard lysis buffer. The cells were then stained with a viability dye and anti-CD3 and anti-PD1 antibodies. Live CD3^+^PD1^+^ T cells were sorted using a BD Aria II cell sorter. The purity of the sorted populations was routinely > 95%. After ligation, these sorted PD1^+^ T cells (1 × 10^5^ PD1^+^ T cells) were injected intramyocardially at five points along the border zone of the infarcted area and the apex of the left ventricle (5 μL per point). The control group received an injection of an equivalent volume of 1640 culture medium. The chest was closed after gently pressing the injection sites with moist gauze for a few seconds. The infarcted mice were placed on a 37 °C warming pad and returned to their cages once they recovered from anesthesia. These sorted PD1^+^ T cells were immediately injected intramyocardially into recipient mice at the time of LAD ligation to assess their functional relevance in post-MI cardiac repair.

### Animal study design

All mice were subjected to permanent ligation of the left anterior descending (LAD) coronary artery to establish an MI model. The animals were randomly assigned to different experimental groups. The total observation period was 28 days. Serial echocardiographic assessments were performed during follow-up to monitor cardiac function. At the study endpoint (Day 28), the hearts were harvested for infarct size quantification, immunofluorescence staining, and protein expression analysis. In experiments involving PD1^+^ T-cell infusion, cells were isolated from the spleens of infarcted mice and immediately injected intramyocardially into the peri-infarct region at the time of LAD ligation. The PD1 inhibitor was administered intraperitoneally at a dose of 200 μg per mouse every other day, starting one day before surgery and continuing until Day 28. IgG was used as an isotype control for the PD1 inhibitor. A CXCR3 inhibitor (300 μg per mouse) was administered starting one day prior to surgery, and this process was repeated every three days throughout the study. Drop-outs occurred because of unexpected intraoperative or postoperative mortality, including intolerance to the surgical procedure, failure to recover from anesthesia, or death before Day 28. These events were not associated with the experimental interventions and were excluded from the final analyses on the basis of pre-defined criteria. The final group sizes used for each analysis are clearly indicated in the figure legends. To minimize bias, the animals were randomly assigned to treatment groups using a computer-generated sequence. Both treatment administration and outcome evaluations (e.g., echocardiography, histology, and molecular analyses) were performed by investigators who were blinded to group allocation. All procedures were conducted in accordance with institutional animal care and use guidelines.

### Establishment of a cynomolgus MI model

The animals were anesthetized via intramuscular injection of ketamine (5 mg/kg) and midazolam (0.2 mg/kg), with ventilation maintained by an animal ventilator and continued gas inhalation-mediated anesthesia. A left lateral thoracotomy was performed, and the heart was exposed using a large animal thoracic retractor. The distal end of the first branch of the left anterior descending coronary artery was ligated with 4–0 silk sutures. Successful MI was indicated by ST-segment elevation after ligation, and the affected cardiac area turned white because of reduced blood supply. This inhibitor nivolumab (10 mg/kg) was intravenously infused one day before and 14 days after MI. Cardiac function was assessed using MRI one day before MI and 3 days and 4 weeks after MI.

### Cardiac MRI

MRI assessments were conducted on a 1.5 T clinical scanner (Siemens) as previously described. The monkeys were anesthetized with intramuscular injections of xylazine (1 mg/kg) and ketamine (10 mg/kg) and positioned supine in the scanner. In accordance with the manufacturer’s instructions, cardiac functional parameters, including the LVEF, ESV, and EDV, were determined using MR myocardial analysis software (Siemens). Infarct size was measured via delayed-enhancement MRI and quantified using the following formula: infarct size% = (scar area volume/left ventricular volume) × 100%.

### Mouse cardiac ultrasound

Echocardiography was performed on all the mice using a Vevo2100 small-animal ultrasound system 1 day before MI modeling and 3, 7, and 28 days post-MI. Ultrasound coupling gel was applied to the ultrasound probe, and the probe was positioned perpendicular to the mouse’s chest plane to obtain 2D images along the long axis of the left ventricle. M-mode echocardiograms were captured at the mid-papillary muscle level in a transverse section. At least five consecutive cardiac cycles were recorded, and parameters such as left ventricular anterior wall thickness, left ventricular posterior wall thickness, LVIDs, and LVIDd were measured. The built-in software was used to calculate the EF, FS, LVSV, and LVDV of the mice.

### Sirius red staining

Heart tissue sections were removed from the −80 °C freezer and rewarmed for 10–15 min. The sections were fixed with 4% paraformaldehyde for 10 min and then rinsed with distilled water. An appropriate amount of Sirius red was applied to each section, and the tissue was mounted using neutral resin. Images were captured with a stereo microscope, and four sections were selected from each heart sample for imaging and statistical analysis.

### WGA staining

Heart tissue sections were fixed with 4% paraformaldehyde for 10 min and then rinsed with PBS. After permeabilization with 0.3% Triton X-100 in PBS for 10 min, the sections were blocked with 5% bovine serum albumin for 30 min at room temperature. The sections were then incubated with fluorescently conjugated WGA working solution at room temperature for 60 min in the dark. After the cells were washed three times with PBS, the nuclei were counterstained with DAPI. The sections were mounted and imaged using a fluorescence microscope. The cardiomyocyte cross-sectional area was quantified from WGA-positive cell borders in randomly selected fields. Quantification was performed in a blinded manner using ImageJ.

### TUNEL staining

The frozen heart sections were rewarmed at room temperature, fixed with 4% paraformaldehyde for 10 min, and then washed with PBS. The sections were permeabilized with PBS containing 0.3% Triton X-100 for 10 min at room temperature, followed by incubation with the TUNEL reaction mixture at 37 °C for 1 h in a humidified chamber protected from light. After being washed with PBS, the sections were blocked with 5% bovine serum albumin for 30 min and then incubated with primary antibody against cTnI at 4 °C overnight. The next day, the sections were washed with PBS and incubated with the corresponding fluorescent secondary antibody at room temperature for 1 h in the dark. The nuclei were counterstained with DAPI. Fluorescence images were acquired using a fluorescence microscope.

### Blood pressure measurement

Blood pressure was measured in conscious mice using a non-invasive tail-cuff blood pressure monitoring system. Systolic blood pressure and pulse rate were recorded at the indicated time points, including baseline before surgery and 3 and 28 days after MI. During measurement, the mice were placed on a warming platform to maintain body temperature and promote tail blood flow. The average value of successful readings was used for statistical analysis.

### Immunofluorescence staining

For immunohistochemistry, frozen tissue sections were fixed in 4% paraformaldehyde for 10 min and permeabilized with PBS containing 0.5% Triton X-100 for 10 min. The sections were then blocked with PBS containing 5% bovine serum albumin and incubated with primary antibodies against PD1 (1:100) and CD3 (1:100) at 4 °C, followed by incubation with the respective secondary antibodies. The nuclei were counterstained with DAPI. Positive-stained cells were imaged and quantified using a Leica fluorescence microscope.

### Isolation and culture of adult and neonatal cardiac fibroblasts

Adult cardiac fibroblasts were isolated from 2- to 3-month-old WT and *PD-L1*^−/−^ mice as previously reported. Hearts were excised, rinsed in cold PBS solution, minced, and digested with type II collagenase (2 mg/mL; Worthington, USA) at 37 °C for 10 min. After digestion, the samples were collected and placed in a neutralizing solution of DMEM supplemented with 10% FBS. The above procedure was repeated 4–5 times, and all the supernatant was collected and centrifuged at 1000 rpm for 5 min. The cells were plated in 12-well plates or 100-mm dishes (Corning, NY) and allowed to attach for 1.5 h before the first medium change, after which weakly adherent cells, including myocytes and endothelial cells, were removed. Fibroblasts were subsequently washed twice with PBS. Rat cardiac fibroblasts were isolated from neonatal mice using the Neonatal Heart Dissociation Kit for Mouse and Rat (Miltenyi, USA).

### scRNA-seq of sham and MI mouse hearts

Single-cell processing of infarcted mouse (7 days post-MI) and sham hearts was performed. Fresh mouse-infarcted hearts were immediately stored in MACS Tissue Storage Solution (Miltenyi Biotech). The tissues were minced and transferred into 5 mL of enzymatic digestion mix consisting of RPMI-1640 medium supplemented with collagenase IV (2 mg/mL) at 37 °C and 100 rpm for 1 h, and this process was terminated by the addition of 5 mL of PBS containing 0.1% BSA. The cells were then filtered through a 70 µm filter and treated with hemolysate buffer for 2–3 min. The cells were subsequently pelleted, washed, and resuspended in FACS buffer.

The single-cell libraries were prepared according to the manufacturer’s instructions for the version 3 (v3) 3’prime kit of the 10X Chromium platform (10X Genomics). The mRNA from each cell was then converted to cDNA through reverse transcription and amplification. This cDNA was subsequently sequenced using an Illumina NovaSeq 6000 to generate raw reads, which typically involves high-throughput sequencing to capture the expression profiles of thousands of cells.

### scRNA-seq data analysis

This resulted in an average read depth of 55,000 reads/cell for the sham group and 54,000 reads/cell for the MI group. Raw reads were aligned to the mouse reference genome mm10 (Ensembl 93) using the 10X Genomics CellRanger (v7.1.0) pipeline. Initial QC and further analysis were performed with the output gene expression matrices using the Seurat (v4.4.0) R package. Low-quality cells expressing fewer than 300 genes, having a UMI greater than 40,000, or having more than 5% of the reads mapped to mitochondrial genes were excluded. Moreover, we used the scDblFinder R package to remove double cells. Data normalization and variance stabilization were performed using the SCTransform method. During SCTransform, cell cycle regression reduces clustering and dimensionality biases driven by proliferative states. The top 3000 variable features identified by SCTransform were used for downstream analysis. The data were subsequently scaled and subjected to principal component analysis (PCA). To correct for batch effects across samples, Harmony (v1.2.0) integration was performed based on the top 50 principal components. Uniform Manifold Approximation and Projection (UMAP) was applied for dimensionality reduction and visualization using the top 50 principal components. Next, the cells were clustered on the basis of gene expression profiles using the Louvain method implemented in Seurat with a resolution parameter of 0.4. After clustering, differentially expressed genes for each cluster were identified using the FindAllMarkers function with the Wilcoxon rank-sum test. Cell-type annotation was performed on the basis of canonical marker gene expression.

T cells were selected from the integrated dataset based on canonical T-cell markers (e.g., Cd3d, Cd3e, and Trdc). The extracted T-cell population was re-normalized and re-scaled prior to dimensionality reduction using the LogNormalize method. Clustering was conducted using the Louvain algorithm at multiple resolutions, and the optimal resolution was selected on the basis of cluster stability and biological interpretability. T-cell subsets, including naive T cells (Ccr7, Sell), CD4^+^ T cells (Cd4), CD8^+^ T cells (Cd8a, Gzma), Tregs (Foxp3, Il2ra), and γδ T cells (Trdc), were annotated on the basis of established marker genes. Finally, visualization plots were drawn to reveal distinct cell populations using the SCP (v0.5.1) tool.

### CyTOF analysis of the PD1^+^ T-cell ratio in the peripheral blood of acute MI patients

We collected peripheral blood from patients with acute MI at admission and 72 h. Peripheral blood samples were collected (2 mL/sample) into ethylene diamine tetraacetic acid (EDTA)-containing anticoagulation tubes (BD Biosciences) and treated with ammonium-chloride-potassium (ACK) lysis buffer (Solarbio Life Sciences) to remove red blood cells. The cells were stained with metal tag antibodies and further washed with FACS buffer for CyTOF analysis. Through CyTOF analysis, we dynamically analyzed changes in PD1^+^ T-cell proportions in the peripheral blood of MI patients at admission and 72 h.

### Flow cytometry analysis of the heart

After the mice were anesthetized, the hearts were perfused with 20 mL of ice-cold PBS. Cardiac tissue samples from different time points post-MI (3 days, 7 days, and 28 days) were immersed in ice-cold PBS. The tissue was digested with 2 mg/mL IV collagenase at 37 °C and 100 rpm for 20 min and allowed to sit for 1 min, after which the supernatant was collected in a neutralizing solution (PBS containing 0.1% BSA). The remaining tissue was subjected to repeated digestion with 1 mg/mL collagenase 2–3 times. All the supernatants were collected and filtered through a 70-µm filter. The solution was subsequently centrifuged at 300× *g* for 5 min at 4 °C to obtain a single-cell suspension. For Th cell subset analysis, separate cells were stimulated with a leukocyte activation cocktail containing phorbol 12-myristate 13-acetate (PMA) and ionomycin for 4–6 h at 37 °C in a humidified 5% CO₂ incubator. After stimulation, the cells were blocked with an Fc blocker, and flow cytometry antibodies were added to prepare them for staining. A pre-mixed solution of flow cytometry antibodies (cell surface markers) was prepared: anti-CD45, anti-CD3, anti-CD4, anti-CD8, anti-CD44, anti-CD279, anti-LAG3, and anti-CD69. After the cells were fixed and permeabilized, anti-IL-4, anti-Th17A, and anti-IFN-γ antibodies were added to the prepared intracellular marker antibody mixture, and the cells were stained for 30 min. For the Foxp3 analysis panel, the cells were subsequently fixed and subjected to nuclear permeabilization using a Foxp3 transcription factor staining buffer set. After the neutralizing solution was added, the samples were centrifuged at 300× *g* for 4 min at 4 °C. The cells were resuspended in 300 µL of flow cytometry neutralizing buffer and then transferred through a filter into flow analysis tubes for analysis.

### Isolation of single cells from tumor tissue in tumor-bearing mice

Fresh mouse tumors were manually cut up and then transferred into 5 mL of enzymatic digestion mix consisting of RPMI-1640 medium supplemented with collagenase IV (2 mg/mL). The tissue digests were processed at 37 °C and 100 rpm for 1 h, and the process was terminated by the addition of 5 mL of PBS containing 0.1% BSA. The cells were then filtered through a 70-µm filter and treated with hemolysate buffer as described previously. Finally, the cells were pelleted, washed, and resuspended in 300 µL of PBS containing 0.1% BSA.

### Fibroblast co-culture with T cells

Neonatal mouse fibroblasts or adult fibroblasts were pre-seeded in 24-well plates (5 × 10^4^ cells/well) and cultured in low-glucose complete medium. The fibroblast medium was changed to low-glucose serum-free medium, and specific inhibitors, the PD1 inhibitor (10 µg/mL) or CXCR3 inhibitor (10 µg/mL), were added as needed for 24 h. Then, the T cells sorted from the infarcted mouse by flow cytometry (2.5 × 10^4^ cells/well) were seeded in the upper chambers of another 24-well plate and cultured in complete RPMI-1640 medium for 2 h. The upper chambers were transferred to the fibroblast-containing 24-well plate for co-culture for 48 h. The cell condition in the lower chambers was observed under a microscope, and after the treatment period, cell protein was harvested, or staining images were taken.

### Cytokine array-based detection of T cells in the MI tissue of mice

PD1^+^ T and PD^-^ T cells from the hearts of mice after MI were sorted using a flow cytometer. After an appropriate amount of RIPA lysis buffer was added, protein quantification was performed, followed by cytokine array detection to analyze inflammatory factors.

### T-cell bulk RNA-seq in mouse MI tissue

RNA-Seq (quantification) uses next-generation high-throughput sequencing technology to analyze eukaryotic gene expression patterns by determining transcription product sequences and performing comparative analysis. Using a BD flow cytometer, PD1^+^ T cells and PD1^−^ T cells were sorted from the infarcted heart, and the sorted cells were centrifuged at 400× *g* for 5 min. Then, 1 mL of TRIzol was added, and the samples were quickly placed in liquid nitrogen. A specific amount of total RNA was extracted for PCR amplification to obtain sufficient double-stranded cDNA for library construction and sequencing. After quality control of the library, sequencing was conducted using the Illumina HiSeq 4000 platform, with read lengths of 2 × 150 bp (PE150).

### Bulk RNA-seq data analysis

After sequencing, raw read quality control was performed with the FastQC (v0.11.9) tool to assess the integrity of the data. The high-quality reads were aligned to a reference transcriptome using HISAT2 (v2.2.1). Next, quantification was performed using the HTSeq (v2.0.3) tool to count the reads associated with each gene. Differential expression analysis was conducted with the DESeq2 (v1.22.2) package to identify genes whose expression significantly differed between PD1^+^ T cells and PD1^−^ T cells. Finally, image production and functional analyses were performed using R packages such as ggplot2 (v3.5.0), pheatmap (v1.0.12), enrichplot (v1.20.0), and clusterProfiler (v4.8.1) to interpret the results of gene characteristics and biological pathway identification.

### Clinical research

With the approval of the Medical Ethics Committees of the Second Affiliated Hospital of Zhejiang University and Hangzhou First People’s Hospital and with informed consent from the patients, inpatients from the cardiology department diagnosed with acute MI, whose angiography indicated at least one coronary artery with a stenosis of ≥ 50% and who were scheduled for stent implantation, were selected. Healthy individuals and patients with coronary heart disease were also included as research subjects. Peripheral blood samples were collected from the patients above to analyze the expression level and proportion of PD1-positive T cells.

### Human heart tissue samples and ethics statement

All the subjects were duly informed, and written consent was obtained from the patients or their relatives. All the studies were approved by the Ethics Review Committee of the Second Affiliated Hospital of Zhejiang University. Left ventricular myocardium samples were obtained from the explanted heart of post-MI patients and healthy donors.

### Statistical methods

The measurement data used in the experiments are expressed as the mean ± standard error of the mean (SEM). GraphPad Prism (v8.0) software was used for data visualization, and SPSS software was used for statistical analysis. Student’s *t*-test was used to compare two groups, while one-way ANOVA was used to compare multiple groups. A *P*-value of < 0.05 was considered to indicate statistical significance.

## Supplementary information

Supplementary Information

## Data Availability

The data that support the findings of this study are available from the corresponding author upon reasonable request. Bulk RNA-seq raw data are available from the GEO database (GSE292863), while scRNA-seq data associated with this study are available from the GEO database under accession number GSE293489.
