# Supplementary material for: Blocking PD1 prevents T-cell-promoted fibrosis in myocardial infarction
Source: Cell Discov. 2026 Oct 6;12:69. doi: 10.1038/s41421-026-00924-2 (PMC13642913; doi:10.1038/s41421-026-00924-2)
Supplement: Supplementary file 1 [file 41421_2026_924_MOESM1_ESM.docx]

**Supplementary Materials and Methods**

**Materials and Reagents**

| **Reagent and Resource Antibodies** | **SOURCE** | **DILUTION** |
| --- | --- | --- |
| α-SMA | ab5694 (abcam, US) | 1:5000 |
| PD1 | 84651T (CST, US) | 1:100 |
| PD1 | Ab214421(abcam, US) | 1:100 |
| Fibronectin | ab2413(abcam, US) | 1:1000 |
| Periostin | ab14041 (abcam, US) | 1:1000 |
| Periostin | AF 2955(RD, US) | 1:1000 |
| Collagen I | ab34710 (abcam, US) | 1:1000 |
| CD3 | ab11089 (abcam, US) | 1:100 |
| β-ACTIN | KC-5A08  (KANGCHEN, Shanghai, China) | 1:3000 |

| **Chemicals and Reagents** | **SOURCE** |
| --- | --- |
| CD45 | 561037(BD Pharmingen) |
| CD3 | 560591(BD Pharmingen) |
| CD4 | 561828(BD Pharmingen) |
| CD44 | 612799(BD Pharmingen) |
| IL-4 | 567083(BD Pharmingen) |
| CD25 | 564023(BD Pharmingen) |
| Foxp3 | 15-5773-80(THERMO) |
| K1-67 | 564071(BD Pharmingen) |
| TNF | 566511(BD Pharmingen) |
| GZMA | 17-5831-80(THERMO) |
| TIM-3 | 747624(BD Pharmingen) |
| TIGIT | 744214(BD Pharmingen) |
| CD45 | 561037(BD Pharmingen) |
| CD3 | 560591(BD Pharmingen) |
| CD4 | 561828(BD Pharmingen) |
| CD44 | 612799(BD Pharmingen) |
| CD3 | 100330(Biolegend) |
| CD140a | 135911(Biolegend) |
| CD69 | 104505(Biolegend) |
| CD274 | 124339(Biolegend) |
| PD1 | 135205(Biolegend) |
| lL-17A | 506925(Biolegend) |
| lCOS | 313537(Biolegend) |
| UltraComp eBeads Plus Compensation Beads | 01-3333`-41(THERMO) |
| Leukocyte Activation Cocktail, with BD GolgiPlug  Transcription Factor Buffer Set | 550583(BD Pharmingen)  562574(BD Pharmingen) |
| MS Separation columns | 130-042-201(Miltenyi) |
| Mouse lL-1β Enhanced Sensitivity Flex Set | 562278(BD Pharmingen) |
| Mouse lL-2 Enhanced Sensitivity Flex Set | 562262(BD Pharmingen) |
| Mouse Enhanced Sensitivity Master Buffer kit | 562246(BD Pharmingen) |
| Mouse TNF Enhanced Sensitivity Flex Set | 562336(BD Pharmingen) |
| Ultra-LEAF™ Purified anti-mouse CD3s | 100340(Biolegend) |
| Ultra-LEAF" Purified anti-mouse CD28 | 102116(Biolegend) |
| Cell Activation Cocktail (with Brefeldin A) | 423303(Biolegend) |

| Human Heart donation | Diseases |
| --- | --- |
| 10538451 | MI |
| 11778967 | MI |
| 02829374 | MI |
| 13449299 | MI |
| 12171171 | Healthy |
| 13022337 | Healthy |
| 12332369 | Healthy |
| 12293350 | Healthy |

**Supplementary Figure and Legends**

**Supplementary Fig. S1. PD1 expression in different types of cells**

**a** Uniform manifold approximation and projection (UMAP) plot showing all non-myocytes subsets from sham and MI mice hearts. **b** UMAP showing distribution of different cell types from sham and MI mice hearts. **c** UMAP showing CD45^+^ immune cells from sham and MI mice hearts. **d** Summary score and proportion of Pdcd1 expression in sham and MI mice hearts.

**Supplementary Fig. S2. PD1^+^ T cells express enrich in mouse MI model.**

**a** Flow cytometry histogram quantifying the PD1-positive cells among CD3^+^ T cells, CD4^+^ T cells and CD8^+^ T cells in the mouse heart at sham and days 3,7,28 after MI. n=6-7 in each group. **b-c** Immunohistochemistry (IHC) assessment for PD1(green) and CD3 (red) in the mouse heart of infarcted regions, including sham, and at days 3,7,28 after MI. Statistical data were plotted using GraphPad Prism. Multiple group comparisons were performed using a one-way ANOVA with Tukey’s post hoc test. n=6 in each group. Scale bar=100 μm.

**Supplementary Fig. S3. PD1^+^ T cells enrich in human MI samples**

**a** Flow cytometry histogram of PD1 on CD3^+^ T cells from the peripheral blood of HD (n=14), AS subjects (n=62), CHD subjects (n=116) and STEMI patients (n=90). **b** Dynamic Cytometry by Time-Of-Flight (CyTOF) analysis reveals the PD1^+^T cells in peripheral blood of patients with AMI at admission and after 72h. **c** Representative images of Sirius red staining showing fibrosis degree in human heart from healthy control and infarcted heart respectively. **d** Quantification of fibrosis degree in human heart of healthy control compared to infarcted heart. n=4 in each group. Scale bar=250 μm. **e-g** Flow cytometry histogram showing the percentage of PD1 level on CD4^+^ T cells and CD8^+^ T cells in the human infarcted heart and the paired peripheral blood, as well as the percentage of CD69^+^ PD1^+^ level, the Lag3^+^ PD1^+^ level on CD4^+^ T cells and CD8^+^ T comparatively. **h** The bar graph shows the PD1 ratio both on the CD4^+^ T cells and CD8^+^ T cells in the human infarcted heart compared to the healthy heart.

**Supplementary Fig. S4. Bulk RNA-seq analysis results and expression levels of functional markers in PD1^+^ T cells**

**a** PCA plot of sequencing samples. **b** Volcano plot of differential genes. Genes with |log_2_FC| > 1, P value adj < 0.05 are highlighted (red: upregulated in PD1^+^ group; green: downregulated in PD1^+^ group). **c** Flow cytometry analysis of TOX, PDL2, TIGIT, CTLA4 expression on PD1^+^T cells and PD1^-^T cells from mice MI heart. n = 3 biological replicates. **d** Flow cytometry histogram showing the PD1 level in the infarcted heart at day 7 and the subcutaneous transplantation tumor with lewis cells as well as exhausted (TIM3, LAG3) and activated (ICOS, CD44) T cells markers in PD1^+^ T cells. **e** GSEA plot of ECM-receptor interaction pathway.

**Supplementary Fig. S5. Cardiac function of PD1 null mice and PD1^+^ T cell infusion after MI**

**a** Study design of WT and PD1^-/-^ mice subjected to MI. **b** Representative M-mode images of WT and PD1^-/-^ mice subjected to MI. **c** Representative M-mode echocardiographic images at pre-MI and 28 days post-MI between the Sham, MI and MI+PD1^+^ T cells group. **d** Immunofluorescence staining for WGA (red) in remote zones in 4 groups (sham, MI, MI+PD1^-^ T, MI+PD1^+^ T n = 6-7). Scale bar,100 μm. **e** Statistical analysis was performed using One-way ANOVA with Tukey’s multiple-comparison test. **f** Kaplan-Meier survival curves for MI mice treated with PD1^+^ T, PD1^-^ T and MI alone.

**Supplementary Fig. S6. PD1 exacerbates cardiac function independent of PD-L1 after MI.**

**a, b** Flow cytometry revealed the expression level of PD-L1 in both WT and activated fibroblasts; statistics were plotted in b. **c** Genotyping identification of PDL1 knockout

transgenic mice. **d** Study design of WT and PD-L1^-/-^ mice with or without PD1^+^T cell infusion subjected to MI. **e** Representative M-mode images of WT and PD-L1^-/-^ mice with or without PD1^+^ T cell infusion subjected to MI. **f** CFSE staining tracing the enrichment of PD1^+^ T cells after injection. **g** Immunostaining reflected the distribution of injected PD1^+^ T cells (red) in infarcted regions of the heart. Scale bar=100 μm.

**Supplementary Fig. S7. CXCL9/CXCR3 axis in PD1^+^ T cell mediated cardiac fibrosis**

**a** White light image indicated the morphology of WT and CXCL9 stimulated cardiac fibroblast. Scale bar=10 μm. **b** Study design of WT mice with CXCR3 inhibitor administration or IgG respectively, followed by PD1^+^ T cell infusion post MI. **c** Representative M-mode images of WT mice with CXCR3 inhibitor administration or IgG respectively, followed by PD1^+^ T cell infusion post MI. **d** Bulk RNA-seq analysis of IKBKB, IRF4 and STAT1 in sorted PD1⁻ and PD1^+^ T cells isolated from mouse hearts 7 days post-MI. **e** Western blot analysis of fibrotic markers fibronectin, collagen I, periostin, and α-SMA in fibroblasts coculture with PD1^-^T, PD1^+^T, PD1^+^T+anti-IgG, PD1^+^T with anti-PD1, PD1^+^T with anti-PD1 and CXCR3 inhibitor, n=4; **f** Statistical analysis was performed using one-way ANOVA with Tukey’s multiple-comparison test. **g** Single-cell RNA sequencing analysis of IKBKB, IRF4 and STAT1 in sorted PD1⁻ and PD1^+^ T cells isolated from mouse hearts 7 days post-MI. **h** Single-cell RNA sequencing analysis of IRF4 expression in cardiac PD1^+^ T cells from infarcted hearts 7 days post-MI relative to those from sham-operated controls. **i** qPCR analysis of IRF4 expression levels in PD1^+^ T cells compared to PD1⁻ T cells isolated from mouse hearts 7 days post-MI.

**Supplementary Fig. S8. Flow cytometric analysis of T cell subpopulations following PD1 blockade**

**a** Gating strategy for lymphocyte isolation. **b–f** Bar graphs show the relative proportions (%) of Th1 (among CD4^+^ T cells), Th17 (among CD4^+^ T cells), Treg (among CD4+ T cells), Th2 (among CD4^+^ T cells), and CD8^+^ T cells (among CD3^+^ T cells), in MI mice treated with anti-IgG or anti-PD1 antibody. g Representative images of TUNEL staining from sequential heart sections both in the border zone (upper left) and remote zone (lower left) from anti-IgG and anti-PD1 groups (n=4-6 in each group). h Summary data analysis of TUNEL positivity in the border zone and remote zone was performed using a t-test. i Blood pressure comparison among 3 groups (sham, anti-PD1, and anti-IgG; n = 6 per group) at baseline and on days 3 and 28 post-MI. j Pulse rate comparison among three groups (sham, anti-PD1, and anti-IgG; n=6 per group) at baseline and on days 3 and 28 post-MI. k Immunofluorescence staining for WGA (red) in border zones in 3 groups (sham, anti-IgG, anti-PD1, n=6-8). Scale bar,100μm. l Statistical analysis was performed using One-way ANOVA with Tukey’s multiple-comparison test. m Kaplan-Meier survival curves for MI mice treated with anti-IgG or anti-PD1.

**Supplementary Fig. S9. Anti-PD1 therapy in non-human primates after MI**

**a** Flow cytometry conducted on isolated CD3^+^ T cells from rhesus monkey with anti-PD1 inhibitor administration or anti-IgG injection post MI, respectively. **b** Inflammatory cytokine array detected from peripheral blood on monkeys with MI 28d in both anti-PD1 inhibitor and anti-IgG group. **c-d** Consecutive monitoring of liver function revealed by ALP and ALT measurement in monkeys with anti-PD1 inhibitor or anti-IgG injection post MI, respectively. **e-f** Consecutive monitoring of renal function revealed by serum BUN and creatine measurement in monkeys with anti-PD1 inhibitor or anti-IgG injection post MI, respectively. **g-h** Consecutive monitoring of thyroid function revealed by FT3 and FT4 evaluation in monkeys with anti-PD1 inhibitor or anti-IgG injection post MI, respectively. n=6 in each group. **i** Systolic blood pressure analysis before and after MI in cynomolgus monkeys between Nivolumab and control groups (n=6 per group). **j** Diastolic blood pressure analysis before and after MI in cynomolgus monkeys between Nivolumab and control groups (n=6 per group).
